# Biotechnological potential and initial characterization of two novel sesquiterpene synthases from Basidiomycota *Coniophora puteana* for heterologous production of δ-cadinol

**DOI:** 10.1186/s12934-022-01791-8

**Published:** 2022-04-19

**Authors:** Marion Ringel, Nicole Dimos, Stephanie Himpich, Martina Haack, Claudia Huber, Wolfgang Eisenreich, Gerhard Schenk, Bernhard Loll, Thomas Brück

**Affiliations:** 1 Werner Siemens Chair of Synthetic Biotechnology, Department of Chemistry, Technical University of Munich, Lichtenbergstr. 4, 85748 Garching, Germany; 2 Institute for Chemistry and Biochemistry, Structural Biochemistry Laboratory, Freie Universität Berlin, Takustr. 6, 14195 Berlin, Germany; 3 Bavarian NMR Center - Structural Membrane Biochemistry, Department of Chemistry, Technical University of Munich, 85748 Garching, Germany; 4 School of Chemistry and Molecular Biosciences, The University of Queensland, 68 Cooper Rd, Brisbane, 4702 Australia

**Keywords:** δ-cadinol, Sesquiterpene, Basidiomycota, Terpene synthases, Active site architecture, Mutagenesis

## Abstract

**Background:**

Terpene synthases are versatile catalysts in all domains of life, catalyzing the formation of an enormous variety of different terpenoid secondary metabolites. Due to their diverse bioactive properties, terpenoids are of great interest as innovative ingredients in pharmaceutical and cosmetic applications. Recent advances in genome sequencing have led to the discovery of numerous terpene synthases, in particular in Basidiomycota like the wood rotting fungus *Coniophora puteana*, which further enhances the scope for the manufacture of terpenes for industrial purposes.

**Results:**

In this study we describe the identification of two novel (+)-δ-cadinol synthases from *C. puteana,* Copu5 and Copu9. The sesquiterpene (+)-δ-cadinol was previously shown to exhibit cytotoxic activity therefore having an application as possible, new, and sustainably sourced anti-tumor agent. In an *Escherichia coli* strain, optimized for sesquiterpene production, titers of 225 mg l^−1^ and 395 mg l^−1^, respectively, could be achieved. Remarkably, both enzymes share the same product profile thereby representing the first two terpene synthases from Basidiomycota with identical product profiles. We solved the crystal structure of Copu9 in its closed conformation, for the first time providing molecular details of sesquiterpene synthase from Basidiomycota. Based on the Copu9 structure, we conducted structure-based mutagenesis of amino acid residues lining the active site, thereby altering the product profile. Interestingly, the mutagenesis study also revealed that despite the conserved product profiles of Copu5 and Copu9 different conformational changes may accompany the catalytic cycle of the two enzymes. This observation suggests that the involvement of tertiary structure elements in the reaction mechanism(s) employed by terpene synthases may be more complex than commonly expected.

**Conclusion:**

The presented product selectivity and titers of Copu5 and Copu9 may pave the way towards a sustainable, biotechnological production of the potentially new bioactive (+)-δ-cadinol. Furthermore, Copu5 and Copu9 may serve as model systems for further mechanistic studies of terpenoid catalysis.

**Graphical Abstract:**

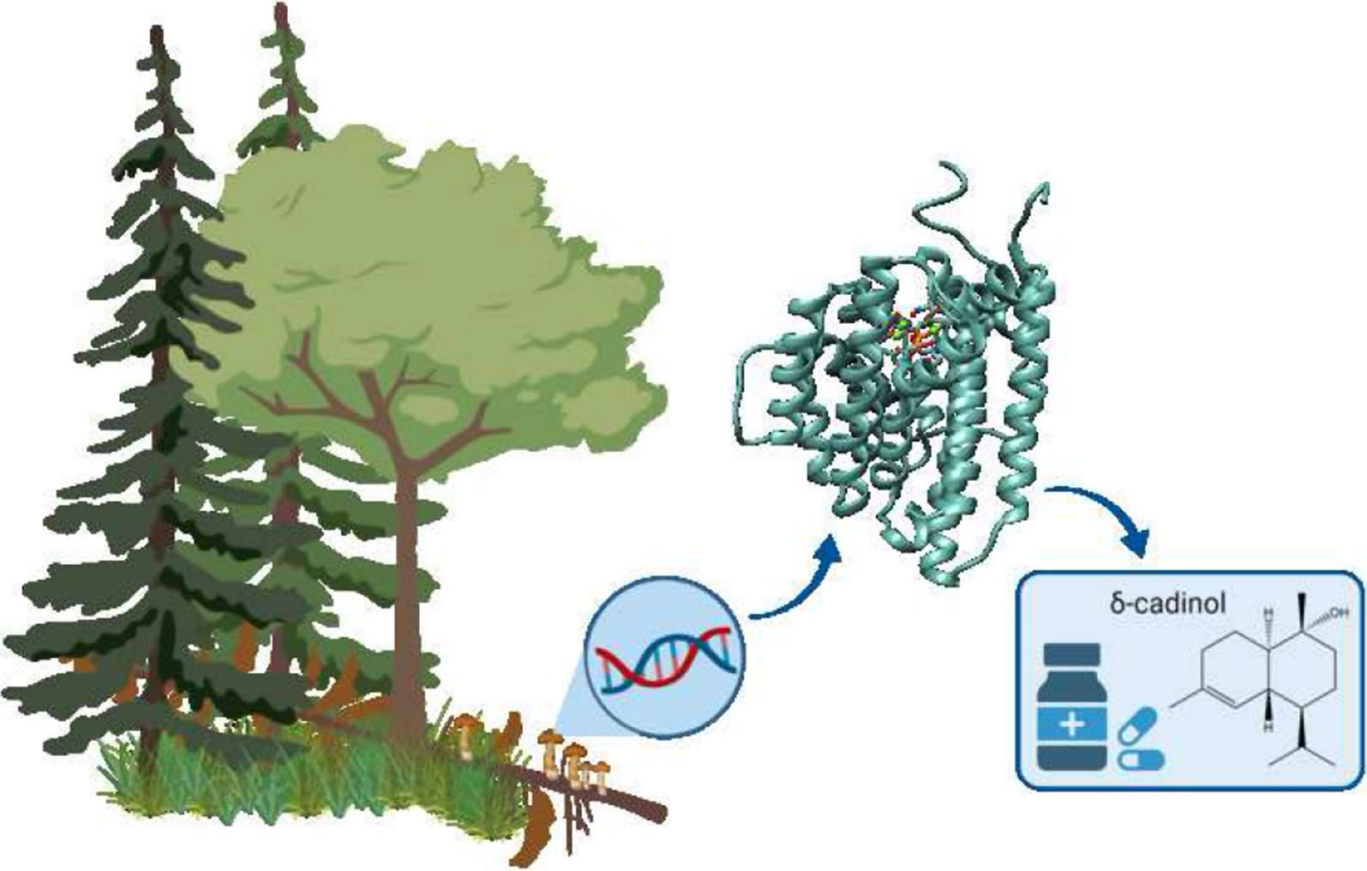

**Supplementary Information:**

The online version contains supplementary material available at 10.1186/s12934-022-01791-8.

## Introduction

The rapid emergence of new diseases (e.g., Covid-19) and the excess use of common drugs such as antibiotics continuously forces the scientific community towards the development of new, innovative drug leads. Over the past decades filamentous fungi have mainly been subject to research focused on the biosynthesis of major antibiotic agents, whereas the identification of bioactive terpenoids was largely based on the analysis of secondary metabolites of medicinal plants [1]. Little attention has been paid to filamentous fungi as potential source of new terpenoid-based bioactives. To date terpenoids represent the largest and structurally most diverse group of natural products encompassing over 80,000 characterized compounds [2]. Terpenoids (and in particular the sesquiterpenoid subfamily) are widely used in medicine and health care for their anti-insect, anti-inflammatory, anti-viral, anti-malarial, anti-microbial and anti-tumor activities [3, 4]. A prominent example for a clinically relevant sesquiterpenoid bioactive is artemisinin, which is a first line treatment against malaria [5]. All sesquiterpenoids feature a complex C_15_ carbohydrate skeleton which is formed by the cyclization of the universal, aliphatic precursor farnesyl diphosphate (FPP), a reaction catalyzed by enzymes from the sesquiterpene synthase family. Sesquiterpene synthases typically belong to the class I terpene synthase (TPS) family, commonly exhibiting a α, αβ or αβγ domain architecture for monofunctional enzymes, with the catalytic site located in their respective α-domains. Bifunctional class I TPS also exist, exhibiting additional catalytic functions in either a second α- or the γ-domain (displaying the class I—class I αα or class I—class II αβγ domain architecture) [2]. All class I TPSs share highly conserved sequence motifs in their respective α-domains, such as the aspartate-rich **D**DXX**D** (DD) dyad and the (**N**,**D**)D(L,I,V)X(**S**,**T**)XXX**E** (NSE) triad, with both being involved in complexing of three Mg^2+^ ions that are essential for catalysis [2]. Furthermore, class I TPSs share a WxxxxxRY sequence motif that facilitates the closure of the active site via salt bridge formation upon substrate binding [6, 7]. In this regard, the *Streptomyces*-derived class I di-TPS CotB2 [8] and the trichodiene synthase from *Fusarium sporotrichioides* [9], yielding the sesquiterpene trichodiene, belong to the best studied TPSs to date [6, 10]. Detailed computational studies on the mechanism employed by trichodiene synthase have highlighted the relevance of a bifacial active site architecture consisting of a highly polar region to promote binding of diphosphate (PP region) and a hydrophobic pocket lined with aromatic amino side chains to guide the propagation of carbocations [10, 11]. Upon the initial substrate binding within the PP region, which also involves the complexation of the diphosphate moiety by the tri-Mg^2+^-cluster, the active site is closed (induced fit) [7, 10]. In the first step of the chemical reaction, C–O bond cleavage and the abstraction of the PP moiety result in the formation of a farnesyl carbocation intermediate. Subsequently, the carbocation relocates to the hydrophobic pocket; the rate of this rearrangement, and therefore the maturing of the carbocation to the respective cyclized (sesqui-)terpene, is controlled by electrostatic interactions within the active site [11]. The structure of the mature terpene is ultimately defined by the amino acid residues lining the hydrophobic binding pocket [2, 11]. Therefore, detailed knowledge about catalytically essential residues within the active site of TPSs may guide the engineering of such enzymes towards targeted products (e.g., new natural bioactives).

This study reports the identification of two new sesqui-TPSs, Copu5 and Copu9, from the wood rotting fungus *Coniophora puteana* via a genome mining approach. Both enzymes can be classified as class I terpene synthases and catalyze the formation of (+)-δ-cadinol as main product from its direct precursor FPP. (+)-δ-cadinol, which is also known as “torreyol” or “pilgerol” [12], has been subject to numerous studies over the past decades including bioactivity tests [13, 14]; plant extracts containing cadinene-type sesquiterpenes (e.g., δ-cadinol) were shown to have anti-microbial, anti-fungal and anti-inflammatory properties [15, 16]. Furthermore, purified (+)-δ-cadinol exhibited cytotoxic activity against MCF7 cells with an IC_50_ of 3.5 ± 0.58 µg ml^−1^ [17]. To date only two other terpene synthases predominantly forming δ-cadinol have been identified, BvCS from *Boreostereum vibrans* [18] and GME3638 from *Lignosus rhinocerotis* [17] with sequence similarities of 41.4% and 58.3% compared to Copu9, respectively (Additional file 1: Fig. S3). Interestingly, exactly like Coniophora puteana, both organisms belong to the division of Basidiomycota. Analyses of the amino acid sequences and corresponding tertiary structure elements of Copu5 and Copu9, employing protein crystallization and homology modelling techniques, revealed that both enzymes share an almost identical active site architecture. Structure-based mutagenesis was employed to probe the role of catalytically important residues, with a view to provide a platform for product-targeted engineering of TPSs.

## Results and discussion

### Identification of potential sesquiterpene synthases

A previous study reported the identification and functional characterization of several putative TPSs within the genome of the wood rotting fungi *C. puteana*. Two of these candidates were indeed shown to be efficient and highly selective sesqui-TPSs producing cubebol and β-copaene, respectively [19]. However, the majority of these putative TPSs still await characterization. To gain further insight into the terpenom of *C. puteana*, the genome of *C. puteana* was probed for the presence of additional TPS-like sequences using a Basic Local Alignment Search Tool (BLAST) with the amino acid sequence of the recently identified cubebol synthase Copu3 [19] as reference. Six candidates were identified (Copu5: XP_007765330, Copu6: XP_007773189, Copu7: XP_007767204, Copu9: XP_007765560, Copu10: XP_007766266.1 and Copu11: XP_007767169.1), all of which contain the aspartate-rich DDXXD motif and the NSE triad, which provide ligands for the three essential Mg^2+^ ions in the active site (Additional file 1: Fig. S1 and S2) [2, 7]. The class I TPS-specific WxxxxxRY motif, a component of the induced fit mechanism, is also conserved [6]. Beyond these motifs the level of sequence conservation is considerably lower, with the six newly identified putative TPS sequences sharing only 24–37% sequence similarity with Copu3.

### Heterologous expression in *Escherichia coli* and characterization of produced sesquiterpenes

**Fig. 1:**
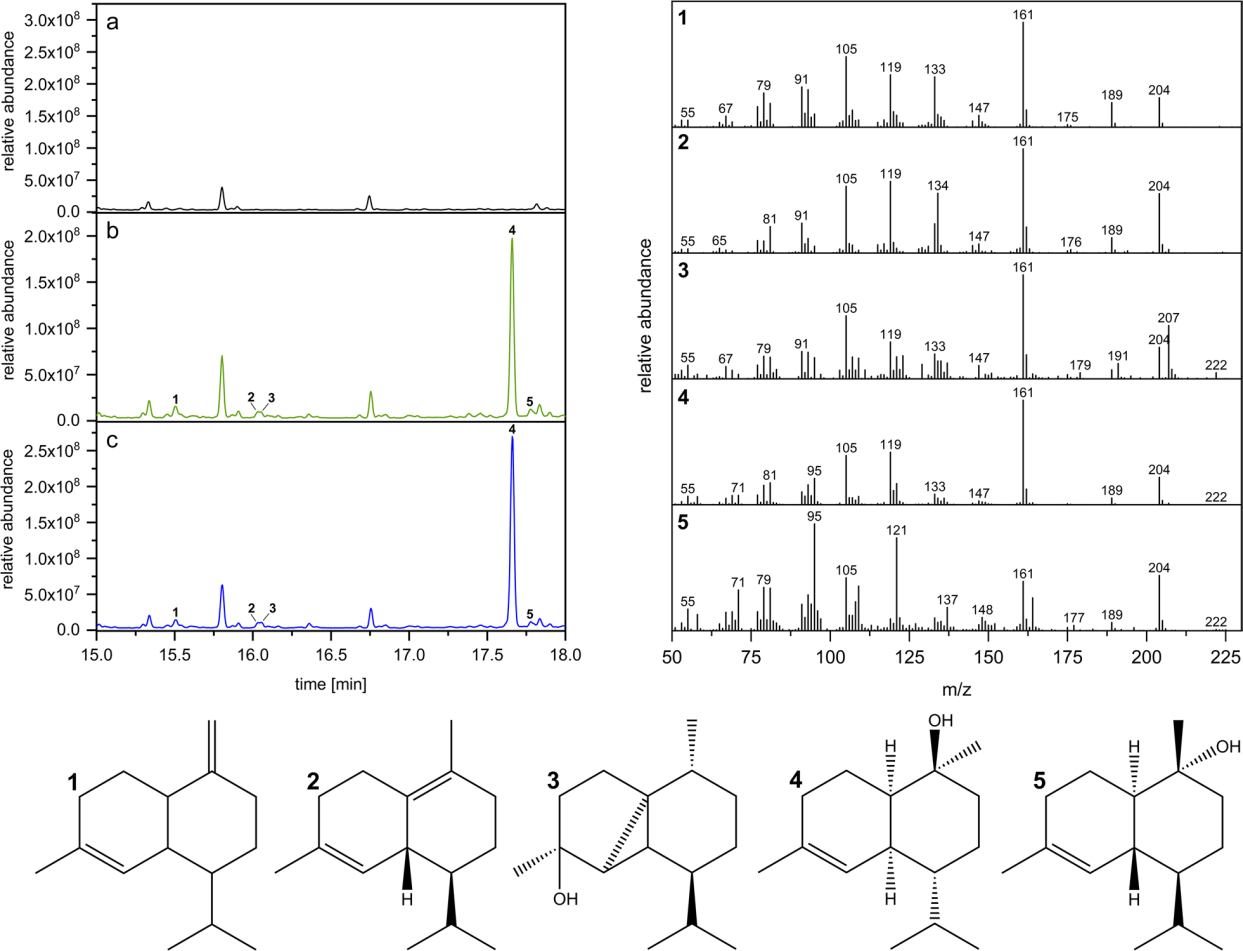
Biosynthetic generation of (+)-δ-cadinol in *E. coli* as whole cell production host; Copu5 (**b**) and Copu9 (**c**) show identical product profiles as shown in the GC chromatograms when compared to a E. coli extract without the biocatalyst as control (**a**). Detailed GC–MS analysis based on NIST database comparisons allows for compound assignment of (1) tau-muurolene (RT: 15.51 min), (2) delta-cadinene (RT: 16.03 min), (3) cubebol (RT: 16.05 min), (4) ( +)-δ-cadinol (RT: 17.66 min) and (5) α-cadinol (RT: 17.78 min)

In order to biochemically and functionally characterize the novel TPSs from *C. puteana*, their respective open reading frames (ORFs) were codon-optimized for heterologous expression in *E. coli* and cloned into a single operon expression system as described previously[19]. The employed expression system, also includes the native *E. coli* non-mevalonate pathway (MEP) bottleneck enzymes 1-deoxy-d-xylulose-5-phosphate synthase (DXS; WP_099145004.1) and isopentenyl-pyrophosphate isomerase (IDI; AAC32208.1) to enhance sesquiterpene production as previously described [19, 20]. In addition, the ORFs of the TPS candidates were also cloned into a two-plasmid diterpene production system, which includes a geranylgeranyl diphosphate synthase (crtE from *Pantoea ananatis*; ADD79325.1) [21], to test for possible catalytic activity towards diterpene production. In preliminary experiments Copu6, Copu7, Copu10 and Copu11 did not show any catalytic activity when tested for sesquiterpene production using the single operon expression vector nor for diterpene production applying the two-plasmid system. Therefore, these four putative TPSs are considered as non-viable protein sequences. Basidiomycota like *C. puteana* are prone to alternative splicing events which especially occur in organisms under stress conditions [22, 23]. Therefore, it is likely that the in silico annotated TPS coding sequences encompass variations of the enzymes, that result in inactive enzyme variants. Active variants of the annotated genes might occur in vivo during splicing events caused by exogenous stress conditions. In contrast, GC–MS analysis of Copu5- and Copu9-expressing *E. coli* extracts showed catalytic activity towards the production of five different sesquiterpenes, when co-expressed with the MEP bottleneck enzymes (Fig. 1). Both cell extracts show typical fragmentation patterns at 105, 119, 161 and 204 m*/z* indicative of non-functionalized, cyclic sesquiterpenes. Moreover, *m/z* values at 105, 119, 161, 204 and 222 suggest that the generated sesquiterpenes are decorated with a single hydroxyl group. Interestingly, Copu5 and Copu9 appear to be very selective showing only one prominent GC–MS signal with a typical mass pattern representative of a mono-hydroxylated sesquiterpene (parent ion 222 m*/z*; RT: 17.09 min). Remarkably, the cyclisation products generated by both Copu5- and Copu9 display the same fragmentation pattern, indicative of formation of identical sesquiterpenes (Fig. 1).

### Structure elucidation of generated sesquiterpenes

In order to structurally identify the sesquiterpenes generated by Copu5 and Copu9 a detailed comparison of their mass spectra with the National Institute of Standards and Technology (NIST) database was performed [24]. This evaluation of the *E. coli* extracts expressing Copu5 and Copu9 revealed (+)-δ-cadinol (RT: 17:66 min; parent ion mass 222 m*/z*, major daughter ions at 119, 161 and 204 m*/z*) as the main product (Fig. 1). The identity of this product was confirmed via NMR experiments (Standardized on solvent (CDCl_3_) peak: ^1^H = 7.26 ppm, ^13^C = 77.2 ppm ^1^H NMR (500 MHz, CDCl_3_) δ 5.51 (dq, J = 5.3, 1.6 Hz, 1H), 1.98 (m, J = 13.9, 10.4, 6.2 Hz, 4H), 1.92–1.85 (m, 1H), 1.66 (s, 3H); 1.63–1.45 (m, 5H), 1.35–1.25 (m, 1H), 1.29 (s, 3H), 1.09 (qd, J = 13.2, 4.2 Hz, 1H), 0.88 (d, J = 6.9 Hz, 3H), 0.81 (d, J = 6.9 Hz, 3H); ^13^C NMR (126 MHz, CDCl_3_) δ 134.36, 124.61, 72.55, 45.55, 44.08, 36.77, 35.31, 31.14, 27.97, 26.41, 23.66, 21.70, 21.52, 18.51, 15.31.) in conjunction with a comparison to reported NMR data (Additional file 1: Figs. S4–S11) [17]. In addition to the main cyclisation product (+)-δ-cadinol, the sesquiterpenes tau-muurolene (RT: 15.51 min), delta-cadinene (RT: 16.03 min), cubebol (RT: 16.05 min) and α-cadinol (RT: 17.78 min) were putatively assigned as minor products in both extracts as indicated by comparison of GC–MS fingerprint spectra with NIST database references (Fig. 1). Based on these product profiles both Copu5 and Copu9 can be designated as new, highly selective (+)-δ-cadinol synthases. The product selectivity of (sesqui-) TPSs varies significantly within this versatile enzyme family ranging from single product formation (e.g., (+)-δ-cadinene synthase from *Gossypium arboreum*) to a product portfolio of over 50 different compounds (e.g., γ-humulene synthase from *Abies grandis*) [19, 25, 26]. In contrast to the common function of an enzyme as an accelerator of a reaction rate, the catalytic challenge for TPSs rather lies in the control of the highly reactive carbocation intermediates alongside their reaction trajectory [27]. The product distribution in TPSs is guided by several factors such as: (i) the activation of the C–O bond by the pyrophosphate-Mg^2+^-cluster in the active site, (ii) electrostatic interactions that lead to the sequestration of the active site, (iii) the specific positioning of water molecules or acidic/basic residues, that facilitate site-specific hydroxylations or (de)protonations, and (iv) a specific active site architecture that pre-shapes the carbocation intermediate [11, 27]. For instance, for two fungal sesquiterpene synthases, Cop4 and Cop6 from *Coprinus cinereus*, it was demonstrated that a smaller carbocation binding pocket lead to a more specific product profile as the carbocation intermediate is more restricted along its potential cyclization routes [28, 29]. At present, all functionally characterized sesquiterpene synthases from *C. puteana* show a highly specific product distribution, indicating that their active site architectures may be very effective in restricting the carbocation intermediates, thereby preventing undesired side reactions.

### Technical scale production of (+)-δ-cadinol

**Fig. 2:**
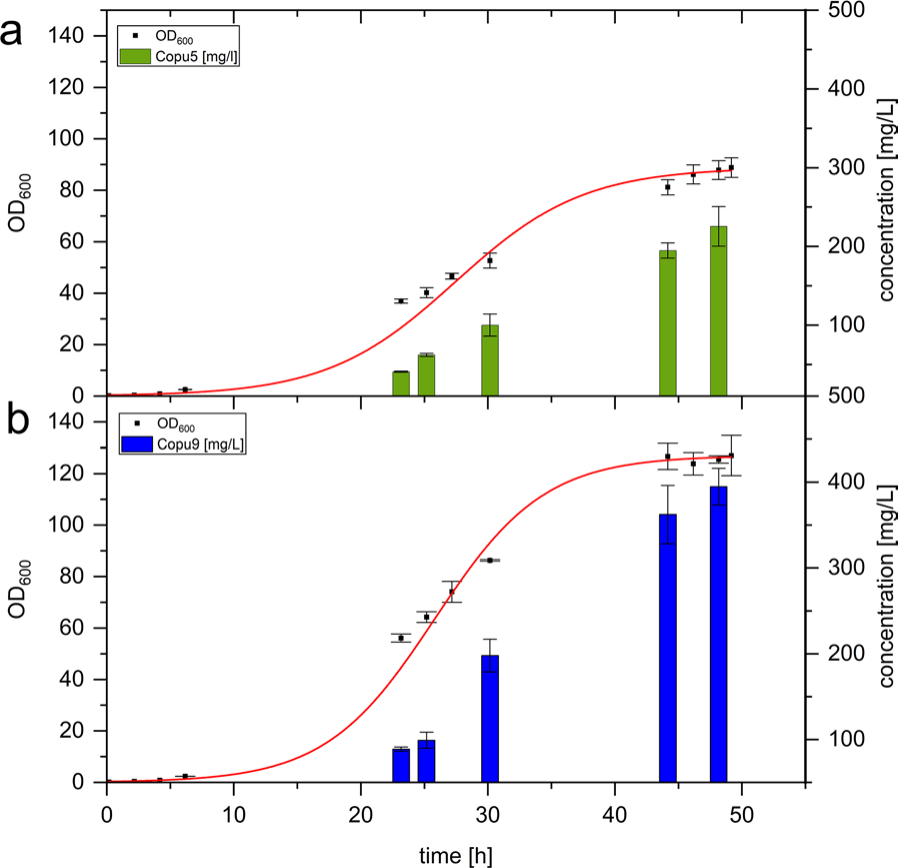
Growth curves of technical scale for the fed-batch fermentation of **a** Copu5 and **b** Copu9 in respective E. coli production hosts and time-dependent (+)-δ-cadinol titers. Error bars represent the mean values ± standard deviation over triplicates

Mischko and co-workers demonstrated that the TPSs from *C. puteana* have both a high product selectivity as well as high product titers [19]. In order to investigate the performance of the newly identified (+)-δ-cadinol synthases Copu5 and Copu9 in an optimized *E. coli* production host, technical scale, fed-batch fermentation experiments were carried out using a 1.3 L parallel fermentation system as described previously [19]. *Escherichia coli* cultures co-expressing Copu5 and the respective MEP bottleneck enzymes reached stationary phase after 48 h with a final OD_600_ of 88 and a (+)-δ-cadinol titer of 225 mg l^−1^ (Fig. 2). Based on this data a Copu5-specific productivity of 4.7 mg l^−1^ h^−1^ was calculated. In contrast, Copu9-expressing cultures reached a final OD_600_ of 126 and a (+)-δ-cadinol titer of 395 mg l^−1^, entering stationary phase after 48 h (Fig. 2). The calculated productivity of Copu9 was 8.2 mg l^−1^ h^−1^, respectively. Since equal fermentation parameters were maintained in the fermentation of Copu5- and Copu9-expressing strains, the only varying factor was the used TPS itself. The resulting different biomass (OD_600_) and δ-cadinol accumulation is thus likely to be a result of a difference in metabolic burden. To date only a few attempts for the biotechnological production of δ-cadinol by microbial hosts have been reported, all of them resulting only in minor yields (no larger than 1 mg l^−1^) [17, 18], and hence, despite its promising pharmaceutical properties, δ-cadinol is mainly referred to as constituent of various plant extracts used in traditional medicine [15, 30, 31]. Therefore, our titers not only significantly exceed those of previous studies, but also, provide an opportunity to sustainably and scalably generate this highly valuable natural bioactive.

### Structural comparison of Copu5 and Copu9

Copu5 and Copu9 exhibit the same product profile (Fig. 1) but differ in their productivities (Fig. 2). The two enzymes share 52.7% sequence identity and a homology of 65.2% (Additional file 1: Figs. S2 and S3). In order to gain insight into residues, that promote the high selectivity of these enzymes, but also the enhanced productivity of Copu9, crystallization trials were carried out. Copu9 could not be crystallized in its open, resting state conformation, but co-crystallization with the non-hydrolysable FPP substrate mimic (4-amino-1-hydroxybutylidene)bisphosphonic acid (alendronate, AHD) and MgCl_2_ resulted in crystals suitable for the collection of X-ray diffraction data. The obtained structure thus represents the closed, catalytically active Copu9 (Copu9·Mg_3_·AHD) conformation. No crystals were obtained for Copu5. In order to further understand the differences of Copu9 and Copu5 a thermal shift assay was performed revealing a significantly lower melting temperature for Copu5 in its apo state as well as bound to AHD [27.6 ± 1.0/33.5 ± 0.8 °C compared to 37.2 ± 0.6/44.3 ± 0.6 °C of Copu9 in pyrophosphate containing Copu5 buffer (Additional file 1 Fig. S15)]. This might be an explanation for the difficulties in purification of Copu5.

**Fig. 3:**
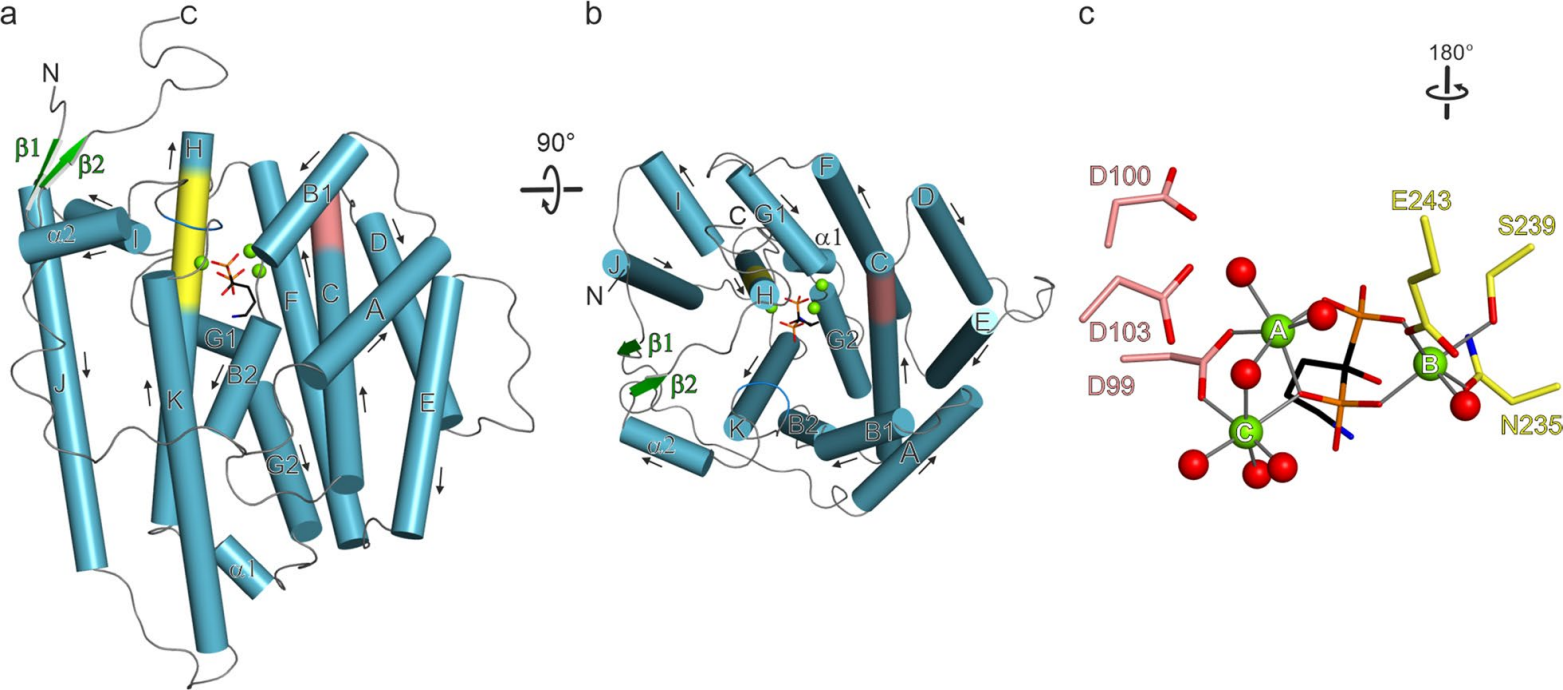
Overall architecture of one monomer of Copu9; **a** α-helices are drawn as blue cylinders and β-sheets as green arrows. The Asp-rich motif is colored in salmon and the NSE motif in yellow. The three Mg^2+^ ions are shown as green spheres and the AHD in stick representation; **b** View of panel a rotated by 90°, resulting in a view from the top into the active site; **c** detailed view of the Asp-rich and the NSE motif. The three Mg^2+^ ions are octahedrally coordinated by side chains of the catalytic motifs, water molecules and the phosphate functions of AHD

Crystals of Copu9·Mg_3_·AHD diffracted to a resolution of 1.83 Å (Additional file 1: Table S1). The enzyme forms a homodimer both *in crystallo* and in solution as observed in size exclusion chromatography. Inspection of the electron density clearly revealed bound AHD and the presence of three Mg^2+^ cations (Additional file 1: Fig. S13) in a closed conformation. Both poly-peptide chains are practically identical with a root mean square deviation (rmsd) of 0.32 Å for 330 pairs of Cα atoms. The structure of Copu9 is complete except for its 13 N-terminal residues. While TPSs generally are helical bundle proteins lacking any β-strands, two short β-strands are present in Copu9, one at the N- and one at the C-terminal ends (Fig. 3a, b and Additional file 1: Fig. S12). These two β-strands (T17-L21 and R335-L339) form an antiparallel β-sheet, which might further stabilize the closed conformation. Copu9 shows the classical ^99^DDWLD^103^ (located on α-helix C) and ^235^NSE^243^ (located on the opposing α-helix H) motifs. The C-terminal ^317^WxxxxxRY^324^ motif adopts a random coil conformation and folds onto the active site, reflecting the closed conformation of Copu9. Latter conformation of the ^317^WxxxxxRY^324^ segment is identical as previously observed in CotB2 [6]. Therefore, both side chains of R223 and Y324 point towards the active site. R223 established a bidentate salt-bridge to Asp99 of the Asp-rich motif. R324 forms a hydrogen bond to one phosphate function of AHD. The pyrophosphate sensor [32] R188 is located on α-helix G1 and establishes a bidentate salt-bridge to the second phosphate group of AHD.

**Fig. 4:**
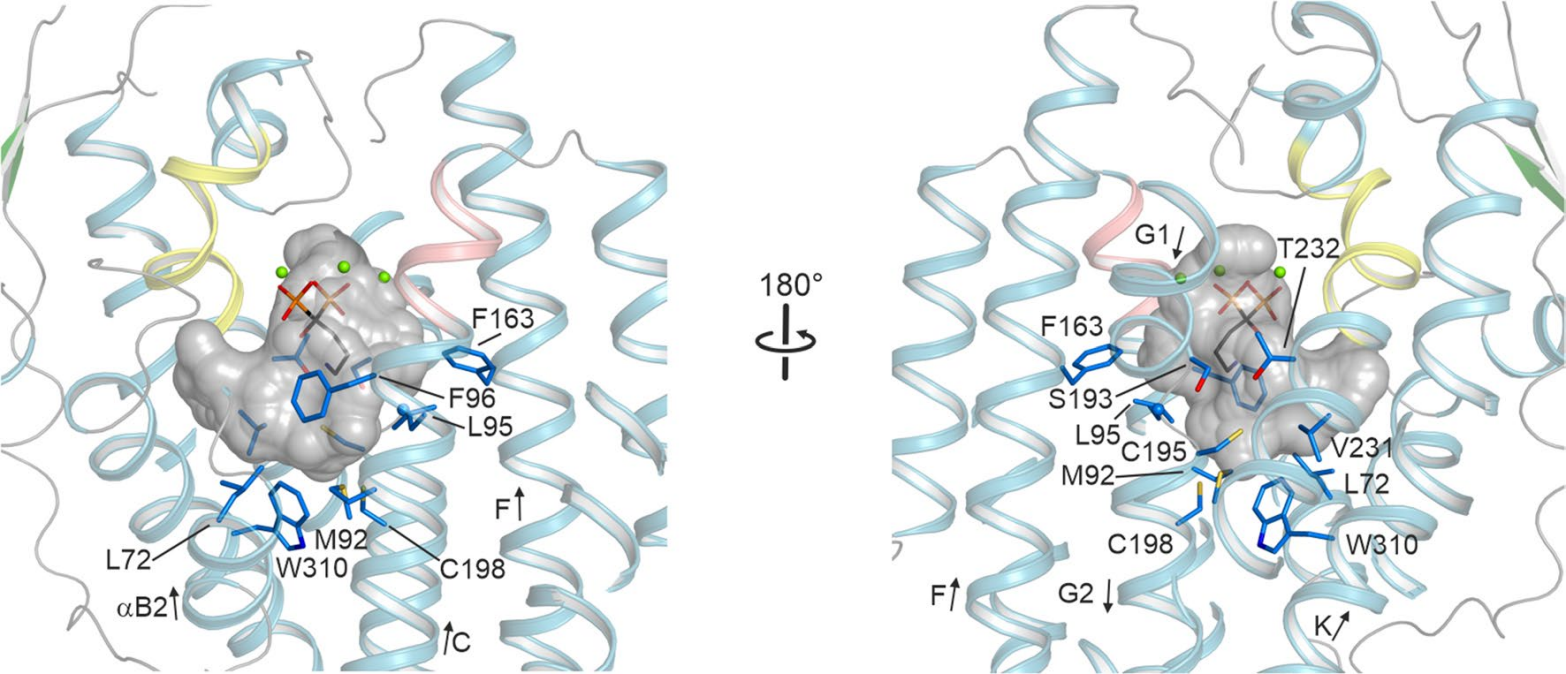
Overall view on residues lining the active site of Copu9. Same color coding as in Fig. 3. The three Mg^2+^ ions are shown as green spheres and the AHD in stick representation. The cavity of the active site pocket is indicated as an inverted surface displayed in gray

The active site is mainly lined by hydrophobic residues: L72, M92, L95, F96, F163, S193, G194, C195, C198, V231, T232 and W310 (Fig. 4). Based on a DALI search [33], the closest structural homologue to Copu9 is Selinadiene synthase (SdS; PDB-ID 4OKM [7]) (Additional file 1: Table S2). The two structures superimpose with a rmsd of 1.45 Å for 296 pairs of Cα atoms.

Since we could not obtain an experimental structure of Copu5, we predicted the structure by the ROBETTA server [34]. To validate the prediction, we initially predicted the structure of Copu9. The obtained model, in its open, inactive conformation superimposes very well, with the experimentally obtained structure of Copu9·Mg2 + 3·AHD (Additional file 1: Fig. S16 and Table S3). The largest differences in the protein backbone are observed in the N- and C-terminal extensions of the protein (Additional file 1: Fig. S16). As anticipated, the active site of the modeled structure is wider, due to the absence of the Mg2+ ions as well as alendronate, since both α-helices harboring the metal binding motifs are tilted away from the active side. A similar observation is made in the model of Copu5 that largely resembles the fold of Copu9 (Additional file 1: Fig. S16b, c and Table S3). The amino acid sequences in their hydrophilic PP binding pockets are highly conserved (DDXXD: Copu5: ^92^DD**W**SD^96^, Copu9: ^99^DD**W**LD^103^; NSE: Copu5: ^227^NDV**F**S**YNK**E^235^, Copu9: ^235^NDI**F**S**YNK**E^243^; WxxxxxRY: Copu5: ^309^W**SF**ETERY^316^, Copu9: ^317^W**SF**DSHRY^324^) (Additional file 1: Fig. S2). Furthermore, the 12 residues involved in either pre-shaping the geometry or the propagation of the carbocations in the hydrophobic pocket of the active site are identical in the two enzymes (Additional file 1: Fig. S12 and Table S4) which is likely to be the cause of their identical catalytic activity. To the best of our knowledge Copu5 and Copu9 are the first reported TPSs from the same organism with the same product profile and an almost equivalent active site decoration. To this end, there are two residues located in the second shell of residues lining the active site and thus merely surrounding first shell residues, which are different between Copu9/Copu5: namely (F91/Y84), and (C198, V190), respectively. By contrast, both Copu5 and Copu9 only share four of the 12 relevant residues in the hydrophobic pocket with the previously reported cubebol synthase Copu3 [19] (Additional file 1: Fig. S12 and Table S4). The remaining eight amino acid side chains are thus likely to play an important role in guiding the product profiles of Copu3 and Copu5/Copu9.

### Structure-based mutagenesis targeting active site residues

**Fig. 5:**
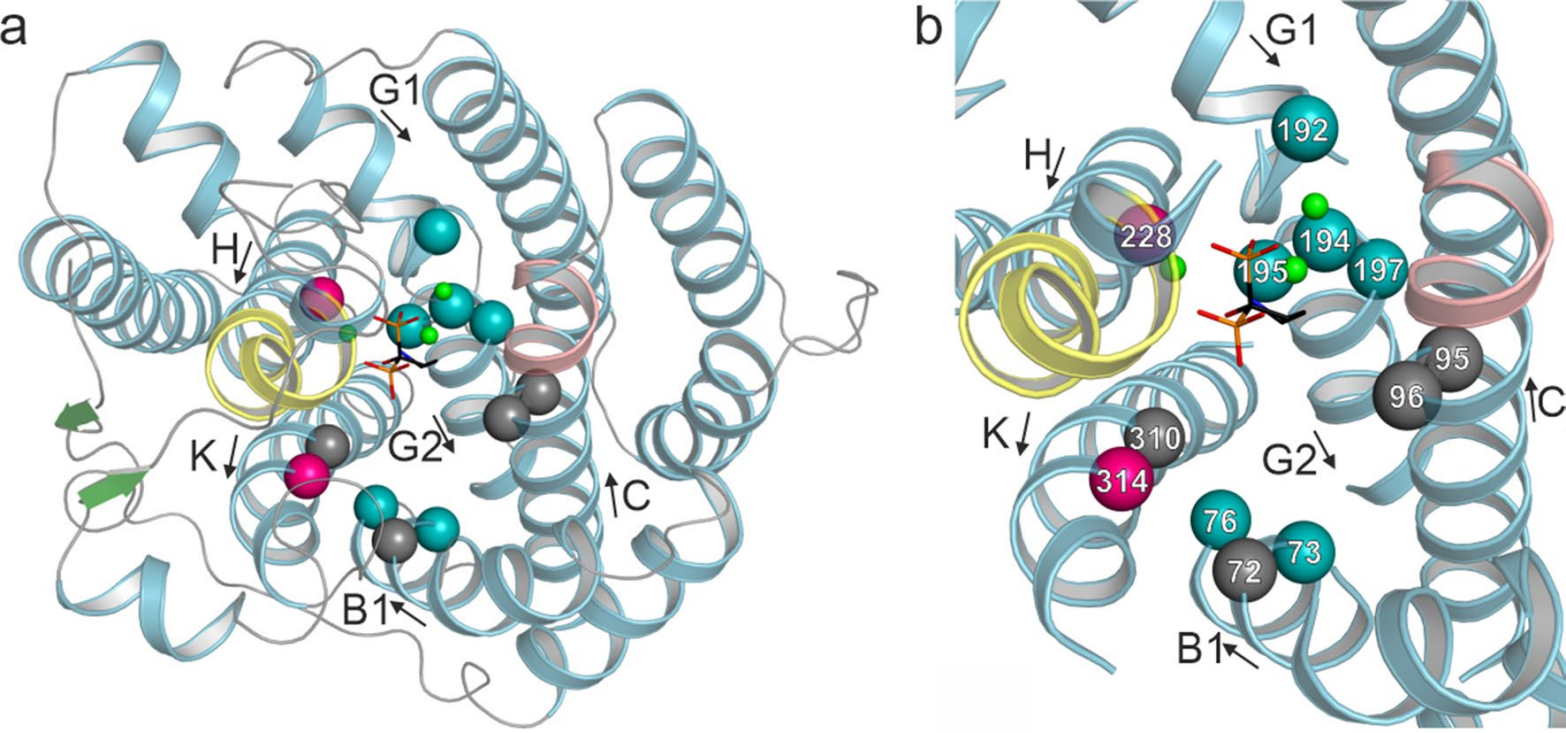
Location of residues subjected to mutagenesis. **a** The same perspective and color-coding as in Fig. 3b were used. α-Helices are drawn as spirals. Identical amino acids between Copu3 and Copu9 are indicated as grey spheres. Residues that have been subjected to mutagenesis without altering the product are shown as teal-colored spheres. Single amino acid exchanges with an effect on the product profile are drawn as magenta-colored spheres. **b** Magnified view into the active site (same perspective and color coding as in panel a), with residues numbered

In order to evaluate their roles, each of them was iteratively changed in Copu5 and Copu9 to their counterpart present in Copu3. As the main product of Copu3 is cubebol [19], which is also produced in minor amounts by Copu5 and Copu9 (Fig. 1), the mutations introduced into Copu5 and Copu9 were anticipated to shift their product profile towards that of Copu3, i.e., generation of cubebol as the main cyclization product (Fig. 5).

The Copu5 variants T66C, C69V and T184N and the Copu9 variants T73C, C76V, T192N, G194A, C195V and P197C did not affect their product spectrum. In contrast, Copu5 variants G186A, C187V and S306N and Copu9 variants N228A and S314N showed increased synthesis of minor products (Fig. 1, Additional file 1: Fig. S17 and S18), while the P189C variant of Copu5 exhibited lower product formation indicating, that this residue is either essential for catalysis or interferes with the catalytically active, closed conformation. Notably, Copu5 variants C187V, N220A and S306N as well as Copu9 variants N228A and S314N showed the formation of an additional side product, germacrene D-4-ol (Additional file 1: Figs. S17–S19; RT: 16.85 min; identified by a comparison to the NIST database [24]). Interestingly, germacrene D-4-ol is also a side product of Copu3.^12^.

**Fig. 6:**
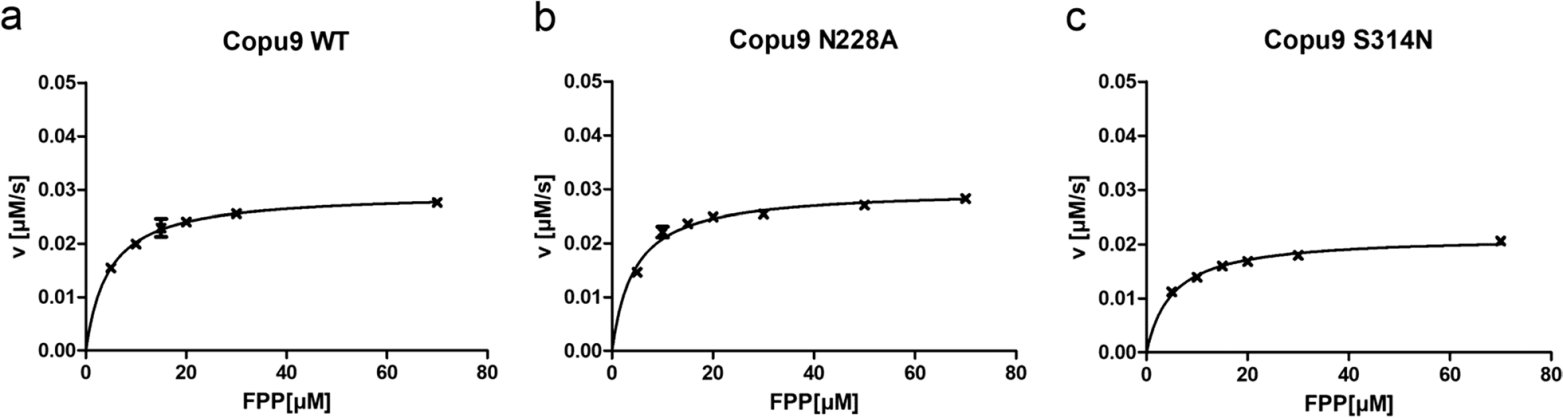
In vitro Michaelis–Menten kinetics of Copu9 WT (**a**) and the variant N228A (**b**) and S314N (**c**) using the EnzChek pyrophosphate Assay. A non-linear regression analysis was performed on the data collected from the time-resolved steady- state kinetic assay

In order to further evaluate the influence of the conducted point mutations on the synthases’ catalytic properties in vitro kinetic experiments were performed. All kinetic parameters obtained from Copu9 and its variants reaction with FPP are listed in Table 1 (Fig. 6, Additional file 1: Fig. S21). Copu9 WT and all variants show comparable binding affinity (K_m_) towards FPP. However, Copu9 variants C76V, N228A and S314N show a minor decrease in catalytic turnover (k_cat_) compared to Copu9 WT, while variants T73C, T192N, G194A, C195V and P197C either retain WT k_cat_ or show a slightly increased catalytic turnover compared to Copu9 WT (Table 1, Fig. 6). Copu9 WT and all variants also show comparable catalytic efficiency (k_cat_/K_m_). However, variant C76V, which showed a minor decrease in catalytic turnover, exhibits slightly higher substrate specificity. Interestingly, the variants N228A and S314N, show a tendency towards decreased substrate specificity which possibly reflects the minor changes in their product spectrum. All kinetic parameters observed for Copu9 WT and its variants are within range of the respective kinetic constants of the kinetically well characterized fungal sesquiterpene synthases Cop4 and Cop6 from *Coprinus cinereus* [29]. In contrast to Copu9, it was only possible to purify Copu5 using pyrophosphate containing buffers due to its significant in-vitro stabilizing effect. Hence, we were not able to determine the in-vitro kinetics of Copu5 and its variants.

**Table 1:** Steady-state kinetic parameters of Copu9 and its variants calculated from the EnzChek™ pyrophosphate assay

Copu9	k_cat_ [s^−1^]	K_m_ [µM]	k_cat_/K_m_ (× 10^3^) [s^−1^ M^−1^]
WT	(3.24 ± 0.03) × 10^–2^	4.59 ± 0.17	7.067
T73C	(5.02 ± 0.26) × 10^–2^	6.28 ± 1.37	7.995
C76V	(2.91 ± 0.07) × 10^–2^	3.14 ± 0.45	9.252
T192N	(3.96 ± 0.08) × 10^–2^	8.28 ± 0.60	4.782
G194A	(3.60 ± 0.05) × 10^–2^	7.00 ± 0.37	5.135
C195V	(3.98 ± 0.07) × 10^–2^	8.48 ± 0.51	4.692
P197C	(3.81 ± 0.10) × 10^–2^	6.82 ± 0.77	5.584
N228A	(2.62 ± 0.07) × 10^–2^	4.42 ± 0.59	5.940
S314N	(1.92 ± 0.05) × 10^–2^	5.10 ± 0.54	3.755

However, none of the variants neither significantly changed the product profile towards another major product, such as cubebol, nor showed a drastic change in its respective catalytic properties. Copu5 appears to be more receptive to single amino acid changes whereas Copu9 largely compensates mutations and retains its wild-type product profile. Recently, it was demonstrated that mutations in loop regions distinct from the active site may result in drastic catalytic differences in related synthases [28]. Considering the proposed cyclization mechanism required to form cadinene-type sesquiterpenes (Additional file 1: Fig. S20) every mutation carried out for Copu5 and Copu9 would be anticipated: (i) to have an effect (stabilizing or destabilizing) on the reaction of the carbocation intermediates as well as, (ii) to allow or restrict water molecule(s) to enter the active site [19, 26, 35]. In particular, residues C187, N220 and S306 in Copu5 and corresponding residues in Copu9 (N228 and S314) appear to have an impact on controlling the carbocation reaction trajectory of the germacryl cation, which is the common intermediate of the cadinene and germacrene cyclization trajectories (Additional file 1:Fig. S20) [19, 26, 29]. However, the impact of single amino acid substitutions in Copu5 and Copu9 is relatively small when compared to other TPSs (e.g., CotB2) [36–39]. In that context, residues lining the entrance of the active site (e.g., residues G186 and C187 in Copu5) affect product specificity.

The directed modulation of a product profile towards a defined product was previously demonstrated for (+)-δ-cadinene synthase from *G. arboreum* [26]. The main product of that synthase was altered towards the production of germacrene D-4-ol by specific mutations within the G1 and G2 α-helices in the hydrophobic pocket of the active site of this enzyme. These helices and the small linker region connecting them, were shown to be important for product specificity in class I TPSs [7, 26, 40]. Upon substrate binding this segment is subject to a conformational change triggered by a conserved effector triad, which was first identified in the C-terminal end of the G1 α-helix of SdS (residues Arg178, Asp181 and Gly182 [7]). This induced-fit mechanism triggers substrate ionization and therefore represents the starting point of the subsequent carbocation cyclization trajectory towards germacrene D-4-ol. By a superimposition of the SdS and Copu9 structures in conjunction with an inspection of a sequence alignment that also includes Copu5, this effector triad could also be identified in the C-terminal ends of the G1 α-helices of Copu5 (R182, D183 and S185) and Copu9 (R190, D191 and S193), respectively. However, instead of the commonly found glycine at the third position of this triad, both Copu5 and Copu9 employ a serine residue (S185 and S193, respectively). Single mutations in the vicinity of the effector triad of Copu9 (i.e., T192, G194, C195 and P197) have no significant effect on the catalytic properties nor product profile of that enzyme (vide supra). However, mutations of the corresponding glycine (G186) and cysteine (C187) residues in Copu5, both located in the linker region between the α-helices G1 and G2, alter the product profile, with the G186A variant also promoting the formation of germacrene D-4-ol (Additional file 1: Figs. S15–S17). This glycine and cysteine residues are the closest residues within the hydrophobic pocket to the phosphate moiety of the bound substrate (see G194 and C195 in Fig. 5).

Therefore, the results presented herein point out: (i) that the serine residue in the effector triad may be important in promoting the production of (+)-δ-cadinol, but also (ii) that there are likely additional tertiary structural elements, that influence the product profile of sesqui-TPSs, possibly by affecting the interface between the hydrophilic and hydrophobic regions in the active site, and/or by interaction with the linker region between α-helices G1 and G2. This is in agreement with studies exploring the influence of domain-domain interactions on the catalytic function of TPSs [41, 42]. It was shown that different bifunctional TPSs exhibit varying degrees of domain-domain interdependence regarding their catalytic activity. Upon separation of the α and βγ domain in Ent-kaurene synthase from *Phaeosphaeria *sp. [42], the single domains still showed catalytic activity but catalytic activity was decreased by 30-fold compared to the wild-type full-length protein. Taxadiene synthase from *Taxus brevifolia* also showed severely compromised catalytic activity in its α-domain when being separated from the βγ-domain [43]. In contrast, the separation of the same domains in abietadiene synthase from *Abies grandis* [41] led to a complete loss of function of the separate domains. Further studies on domain-domain chimeras of fusicoccadiene synthase from *Phomopsis amygdala* as well as ophiobolin F synthase from *Aspergillus clavatus* showed significantly altered cyclization fidelity and catalytic activity compared to the wild-type enzymes [43]. The architecture of multi-domain TPS might contribute to the overall stability of the proteins. Moreover, tertiary structure interactions play a significant role in shaping the active site for precise chemical control along the carbocation reaction trajectory. Especially for residues involved in conformational changes during catalysis or flexible tertiary structure elements this might play a bigger role than hitherto expected.

## Conclusion

The genomes of terrestrial and marine organisms bear an enormous and at present widely uncharacterized capacity of TPSs mediating the formation of natural bioactives [44, 45]. Similarly, although fungi are known for their broad variety of bioactive terpenoids, fungal TPSs have not yet been explored in depth for their potential for biotechnology applications [35]. In this study six novel putative TPSs from the Basidiomycota *C. puteana* were characterized. Only two of them, Copu5 and Copu9, were functional and identified as efficient and highly product selective (+)-δ-cadinol synthases (Fig. 1). Notably, these two synthases are the first TPSs that originate from the same organism and yet have virtually identical product profiles. The observed product selectivity of both synthases is likely to be a results of highly conserved sequence motifs and a carefully and equally decorated active site. Both display excellent production and productivity for cadinol, exceeding currently available biosynthesis systems by far (Fig. 2).

The crystal structure of Copu9 in complex with three catalytically essential Mg^2+^ ions and the substrate mimic AHD was solved to a resolution of 1.83 Å (Fig. 3). This structure is the first of a class I TPS from Basidiomycota. Structure-informed mutations in the hydrophobic pockets of the active sites provided proof-of-concept, that single amino acid changes can alter the product profile of Copu5 and Copu9 from (+)-δ-cadinol to germacrene D-4-ol. However, despite having identical residues in the hydrophobic substrate binding pocket, Copu5 appears to be more flexible towards changing its product profile than Copu9. For Copu5 mutations targeting the linker between the α-helices G1 and G2 affected the product spectrum (Additional file 1: Fig. S12). This structural segment has previously been shown to be important for the product specificity in class I TPSs [7, 26, 40]. To this end, the herein presented results in conjunction with the results previously shown for a (+)-δ-cadinene synthase [26] suggest that the helix-turn-helix motif may be a hotspot for product shifting mutations and thus serve as a guiding significance for the improvement of related class I terpene synthases. The question remains why Copu9, unlike other members of this class of enzymes, appears to be less affected by mutations in this region. This observation may suggest a structural robustness of Copu9 and may also be connected to its high product yield and specificity when expressed in *E. coli*. It is likely, that additional structural elements and associated conformational changes play an important role in modulating the catalytic performance and product profile of class I TPSs. In this respect, Copu5 and Copu9 present ideal model systems to enhance mechanistic insight into this important class of enzymes for applications in biotechnology and synthetic biology.

## Experimental section

### General

All media components and HPLC grade chemicals were purchased from Roth chemicals (Karlsruhe, Germany). Technical grade solvents were obtained from Westfalen AG (Münster, Germany). CDCl_3_ and Benzene-d_6_ were purchased from Sigma-Aldrich (St. Louis, USA).

### Gene cloning, plasmid construction and culture condition

*Escherichia coli* strain DH5α was used for all cloning steps and *E. coli* strain HMS174 (DE3) for terpene production. Genes encoding putative TPSs Copu5 (XP_007765330), Copu6 (XP_007773189), Copu7 (XP_007767204), Copu9 (XP_007765560), Copu10 (XP_007766266.1) and Copu11 (XP_007767169.1) from *C. puteana* were codon-optimized for *E. coli* using the GeneOptimizer™ software and subsequently synthesized by Eurofins Genomics GmbH (Ebersberg, Germany). For sesquiterpene production all genes were cloned into a pACYC-based vector system containing a single operon with selected bottleneck enzymes of the MEP pathway as previously described [19] and the respective TPS all set under the control of a *lac*-I-derived constitutive promoter [21]. For determination of potential catalytic activity towards diterpene production all genes were cloned into a pACYC-based expression vector system and co-transformed with a plasmid containing essential bottleneck enzymes for diterpene production as previously described [21]. All cloning experiments were performed according to standard protocols.

Cultures were grown in modified R-Media [46] (13.3 g l^−1^ KH_2_PO_4_, 4.0 g l^−1^ (NH_4_)_2_HPO_4_, 1.7 g l^−1^ citric acid, 5.0 g l^−1^ yeast extract, 4.88 ml l^−1^ 1 M MgSO_4_, 2.45 ml l^−1^ 0.1 M Fe(III) citrate, 1.00 ml l^−1^ 100 × Trace Element Solution (5.0 g l^−1^ EDTA, 84 mg l^−1^ ZnCl_2_, 13 mg l^−1^ CuCl_2_*2 H_2_O, 10 mg l^−1^ CoCl_2_*2 H_2_O, 10 mg l^−1^ H_3_BO_3_, 1.6 mg l^−1^ MnCl_2_*4 H_2_O) and 30 g l^−1^ glycerol at 30 °C and 100 rpm shaking. The appropriate antibiotics kanamycin (50 µg ml^−1^), chloramphenicol (35 µg ml^−1^) or ampicillin (100 µg ml^−1^) were added as needed.

### Fermentation

Fermentation on a technical scale was performed using a DASGIP® 1.3 L parallel reactor system (Eppendorf AG, Germany) with a modified R-media as described above. An overnight preculture was used for the inoculation of the fermenters (OD_600_ = 0.1). Cultivation temperature was kept constant at 30 °C. Initial stirring velocity and air flow were set to 200 rpm and 0.2 volumes of air per volumes of medium per minute (vvm), respectively. Dissolved oxygen was kept constant at 30% and maintained by a gradual increase in stirring velocity (max. 1000 rpm), oxygen content (max. 100%) and airflow (max. 0.8 vvm) during fermentation. A pH value of 7.0 was controlled by the addition of 25% aqueous ammonia. A pH-based feeding protocol was set as previously described [19, 21]. The feeding solution consisted of 600 g l^−1^ glycerol, 5 g l^−1^ yeast extract, 35 g l^−1^ collagen, 20 g l^−1^ MgSO4, 0.3 g l^−1^ thiamine-HCl, 5 ml l^−1^ 1 M ammonium iron(III) citrate, 20 ml l^−1^ 100 × trace element solution (pH = 7.0) [19, 47].

### Terpene extraction

To extract terpenes during the screening process 20 ml of the *E. coli* culture broth were mixed with 20 ml of an extraction solution (ethanol, ethyl acetate and hexane; 1:1:1). The mixture was shaken for 4 h at room temperature and subsequently centrifuged down for 5 min at 8000 rpm to separate the organic phase. A sample from the organic phase was then analyzed via GC–MS.

The cultivation broth from either large-scale shaking flask experiments (1 l) or fermentation using a DASGIP^®^ 1.3 l parallel reactor system (Eppendorf AG, Germany) was extracted by adding the same volume of ethanol. This mixture was shaken on a rotary shaker (80 rpm) at 20 °C for 12 h. Subsequently ½ volume of ethyl acetate was added and shaken for 3 h (20 °C, 80 rpm) followed by a centrifugation step for 15 min at 7000*g* to separate the supernatant from the cell debris. Afterwards the ½ volume of hexane was added to the supernatant and the extraction was carried out for 3 h (20 °C, 80 rpm). The organic phase was separated using a separation funnel and subsequently concentrated using a rotary evaporator.

### Terpene purification

The crude extract was evaporated until only the oily resin remained. The resin was dissolved in 10 ml of hexane. Subsequently, flash chromatography was carried out to separate the terpene fraction from fatty acid residues using the flash chromatography system PLC 2250 (Gilson, USA) equipped with a Luna 10 µm silica (2) 100 A column at a flow rate of 10 ml min^−1^. Peaks were detected using an Evaporative Light Scattering Detector (ELSD) flushed with nitrogen gas and a diode array detector at 40 °C. The following gradient was applied: 100% hexane (solvent A) for 15 min, followed by a rapid change (within 3 s) to 100% EtOAc (solvent B), a 15 min wash with solvent B, a return to 100% solvent A within 3 s and a final wash with that solvent for further 30 min. Fractions of interest were reduced to approximately 2 ml using a rotary evaporator and mixed with acetonitrile. Subsequently, the residual hexane was evaporated until only acetonitrile (ACN) remained.

For further purification of the products, the samples were injected into an Ultimate 3000 UHPLC system (Thermo Scientific, USA) containing a binary pump, a diode array detector, an automated fraction collector, and a Jetstream b1.18 column oven. The purification of the respective sesquiterpenes was carried out on a NUCLEODUR® C18 HTec 250/10 mm and guard column holder 8 mm (Machery-Nagel GmbH & Co. KG, Germany) at 30 °C and a flowrate of 2.2 ml min^−1^ using H_2_O and ACN as solvents. The following gradient was applied: 90% ACN for 0.5 min, increased to 100% ACN within 10 min to remain for 12 min, decrease to 90% ACN within 0.1 min to remain for another 10 min. Fractions containing the sesquiterpene of interest were evaporated under low nitrogen flow to dryness and subsequently dissolved in the solvent of interest (hexane for GC–MS analysis or CDCl_3_ for NMR analysis).

### Analytics

Analysis and quantification of terpenes was performed using a Trace GC–MS Ultra system with DSQII (Thermo Scientific, USA). The sample (1 µl, 1/10 split) was injected by a TriPlus auto sampler onto a SGE BPX5 column (30 m, I.D 0.25 mm, film 0.25 µm) with an injector temperature of 280 °C. Helium was used as carrier gas with a flow rate of 0.8 ml min^−1^. Initial oven temperature was set to 50 °C for 2 min. The temperature was increased to 320 °C at a rate of 10 °C/min^−1^ and then held for 3 min. MS data were recorded at 70 eV (EI) in positive mode in a range between 50 and 650. GC-FID analysis was carried out accordingly. Quantification of sesquiterpenes was carried out by correlation of the FID peak area to a defined α-humulene standard of known quantity as previously described [19].

Purified compounds for further NMR analysis were dissolved in CDCl_3_. ^13^C NMR spectra were measured with a Bruker Avance-III 500 MHz spectrometer equipped with a cryo probe head (5 mm CPQNP, ^1^H/^13^C/^31^P/^19^F/^29^Si; Z-gradient). ^1^H NMR spectra as well as 2D experiments (HSQC, HMBC, COSY, NOESY) were obtained on an Avance-I 500 MHz system with an inverse probehead (5 mm SEI; ^1^H/^13^C; Z-gradient). The temperature was set to 300 K. Resulting data were processed and analyzed by TOPSPIN 3.2 or MestreNova 11.0. Chemical shifts were given in ppm relative to CDCl_3_ (δ = 7.26 ppm for ^1^H and δ = 77.16 ppm for ^13^C spectra).

### Protein expression and purification for crystallization experiments

The codon optimized genes encoding Copu5 and Copu9 were fused to an N-terminal hexa-histidine-tag in a pET-M11 vector and transformed into *E. coli* BL21 RIL DE3. Overexpression was performed using auto-induction medium at 37 °C until an OD ~ 0.7 was reached and subsequently cooled down to 18 °C [48]. Cells grew 48 h and were harvested by centrifugation (10 min, 6000 rpm at 4 °C). For resuspension of the cell pellets, buffer A was used (Copu9: 20 mM Tris/HCl pH 7.5, 500 mM NaCl, 5 mM MgCl_2_; Copu5: 100 mM Tris/HCl pH 7.5, 500 mM NaCl, 5 mM MgCl_2_, 10% (w/v) glycerol, 10 mM sodium pyrophosphate, 1 mM DTT). Cells were lysed by homogenization at 4 °C and the lysate was cleared by centrifugation (1 h, 21,000 rpm at 4 °C). Ni^2+^-NTA beads (cv ~ 1 ml; GE Healthcare) were equilibrated with buffer A. Copu9 was loaded on the column and washed with 10 cv of buffer A containing additional 30 mM imidazole. Copu9 was eluted with buffer A containing 250 mM imidazole. Size exclusion chromatography was performed with a HighLoad Superdex S200 16/60 column (GE Healthcare), equilibrated with buffer B [(Copu9: 20 mM Tris/HCl pH 7.5, 150 mM NaCl, 5 mM MgCl_2_; Copu5: 100 mM Tris/HCl pH 7.5, 250 mM NaCl, 5 mM MgCl_2_, 10% (w/v) glycerol, 10 mM sodium pyrophosphate, 1 mM DTT)]. Pooled protein fractions were concentrated with an Amicon-Ultra 30,000 cell. Calibration runs were performed with the high molecular weight standard (GE Healthcare).

### Thermal shift assay

Melting temperatures of Copu9 variants and Copu5 were measured with the Mx3005P qPCR system (Agilent) in a 96-well plate format with and without alendronate. Each well contained 8 µl SEC buffer (either of Copu9 or Copu5, as stated in the manuscript), 10 µl protein (0.15 µg µl^−1^) with 1× SYPRO Orange dye (Invitrogen) end concentration and either 2 µl water or 2 µl alendronate (0.6 mg/ml) dissolved in water. The program consisted of three steps: step 1 was a pre-incubation for 1 min at 20 °C, and steps 2 and 3 were cycles comprising the temperature increase of 1 °C within 20 s. The temperature gradient proceeded from 25 to 95 °C at 1 °C per minute. Samples were measured in triplicates. The data was acquired with MxPro QPCR software (Agilent, Germany) and analyzed with DSF Analysis v3.0.1 tool (ftp://ftp.sgc.ox.ac.uk/pub/biophysics) and Graphpad Prism 5.0.0.228 (Graph Pad Software Inc.). A t-test was performed with Graphpad Prism to validate the significance of the results.

### In-vitro kinetics

Time resolved kinetics were measured using the EnzChek™ pyrophosphate assay kit (Invitrogen™, ThermoFisher Scientific) in a 96-well-plate with a reduced volume of 200 µl. The assay was performed as stated in the manual using 0.8–1.2 µM of the Copu9 variants and FPP (Sigma-Aldrich) concentrations ranging from 0 to 70 µM dissolved in methanol. Everything except the substrate was mixed and preincubated at 25 °C for 10 min. The substrate was added shortly before measurement and mixed for 5 s prior the first measurement. The enzymatic reaction was performed at 25 °C and the absorption was measured at 360 nm in 30 s increments using a CM Spark plate reader (Tecan, Germany). The data was analyzed using Graphpad Prism 5.0.0.228 (Graph Pad Software Inc.).

### Crystallization

For co-crystallization experiments, Copu9 was concentrated to 28 mg ml^−1^ as measured by the absorbance at 280 nm and incubated with a 10 -fold molar excess of AHD for 30 min on ice. Initial crystals were obtained by the sitting-drop vapor-diffusion method at 18 °C with a reservoir solution composed of 15.0% (w/v) polyethylene glycol 3350, 100 mM Tris/HCl at pH 8.5 and 100 mM Mg formate. Initial, inter-grown crystals were used to prepare a seed stock. With a cat whisker, seeds were transferred to a freshly prepared crystallization drop and crystallization plates were subsequently stored at 4 °C. Prior to flash-freezing in liquid nitrogen, crystals were transferred to a cryo-protectant solution composed of the reservoir solution supplemented with 25% (v/v) ethylene glycol.

### Structure determination and refinement

Synchrotron diffraction data were collected at the beamline 14.1 of the MX Joint Berlin laboratory at BESSY (Berlin, Germany). Diffraction data were processed with XDS [49] (Additional file 1: Table S1). The structure was determined by molecular replacement using the coordinates of SVS_A2 (PDB-ID: 6TJZ [50]) as search model using the PHASER software [51]. The structure was refined by maximum-likelihood restrained refinement in PHENIX Model building [52, 53] and water picking was performed with COOT [54]. Model quality was evaluated with MolProbity [55] and the JCSG validation server (JCSG Quality Control Check v3.1). Secondary structure elements were assigned with DSSP [56] and ALSCRIPT [57] was used for secondary structure based sequence alignments. Figures were prepared using PyMOL (Schrödinger, Inc.). The DALI server [33] was used to identify structures closely related to Copu9.

### Protein modelling and phylogenetic analysis

Sequence alignments were performed using Clustal Omega [58] by employing seeded guide trees and HMM profile techniques as previously described [19]. To predict the tertiary structure of Copu5 and Copu9 the ROBETTA server was used [34]. The primary sequence of both proteins was submitted, and the server was run with standard settings. All calculated models have been analyzed and verified by SAVES 6.0 (https://saves.mbi.ucla.edu/) which were subsequently analyzed within the UCSF Chimera environment [59, 60]. For further comparison of the sesquiterpene synthases sequences AliView [61] was used.

## Supplementary Information

**Additional file 1.** Additional figures and tables.

## Data Availability

All data generated of analyzed during this study are included in this article and the supporting information. Coordinates and structure factors have been deposited in the PDB (7OFL). Diffraction images have been deposited at proteindiffraction.org (https://doi.org/10.18430/m37OFL).
