# Supplementary material for: Biotechnological potential and initial characterization of two novel sesquiterpene synthases from Basidiomycota Coniophora puteana for heterologous production of δ-cadinol
Source: Microb Cell Fact. 2022 Apr 19;21:64. doi: 10.1186/s12934-022-01791-8 (PMC9018054; doi:10.1186/s12934-022-01791-8)
Supplement: Supplementary file 1 — Additional file 1. Additional figures and tables. [file 12934_2022_1791_MOESM1_ESM.docx]

**Additional file 1**

# Biotechnological potential and initial characterization of two novel sesquiterpene synthases from Basidiomycota *Coniophora puteana* for heterologous production of δ-cadinol

Marion Ringel^1^ ‡, Nicole Dimos^2^ ‡, Stephanie Himpich^2^, Martina Haack^1^ , Claudia Huber^3^ , Wolfgang Eisenreich^3^ , , Gerhard Schenk^4^ , Bernhard Loll^2 *^ and Thomas Brück^1 *^

^1^Werner Siemens Chair of Synthetic Biotechnology, Dept. of Chemistry, Technical University of Munich (TUM), Lichtenbergstr. 4, 85748 Garching, Germany

^2^Institute for Chemistry and Biochemistry, Structural Biochemistry Laboratory, Freie Universität Berlin, Takustr. 6, 14195 Berlin, Germany

^3^Bavarian NMR Center—Structural Membrane Biochemistry, Department of Chemistry, Technische Universität München, 85748 Garching, Germany

^4^School of Chemistry and Molecular Biosciences, The University of Queensland, 68 Cooper Rd, 4702 Brisbane, Australia

## Fig. S1. Amino acid sequences

>Copu3 [1]

MSATPAPTEFILPNLFSVCPLTFGRSNPYYDEVIPEARAWIAKYNPFVDSKRAEFVQGCNELLCSRVWPYAGREEFRTCCDFVNLLFVLDELSDDMGGADARSTCDSFIRVLNDPDAPDTSLIAQMTREFRARVAERAKPGCLRRFIALCGTYVEAVCVEAELREQGRVLDLRSFILLRRENSAVRCCLALAEYALGLELPDAVFNDPAFQSVYFCAADMVCWSNDVYSYNMEQAKGHTGNNVVTVLMQEHGIDLQAAADRVGEVFGQLMEHYTSGSRSLPTWGGKVDADAARFLEAAGQWVVGNLEWSFETPRYFGPDHDEVRDTHRVLLK

>Copu5

MHLPEPFHFLLPDFSSHCSYPLRLNKHCVAAAAASEDWLIRLAQLRSPRNGRKLKKFMGLKAGYLTALCYPDCPRTELRVVSDYMNFLFTLDDWSDEFAEAGVRGLEQCVMGMLYDPTVKTDKAAGRLARSFWLRMIRTAGPRVQHRFIVAFEDFFRAVEQQSRDRAKGVMPDLESYIALRRDTSGCRPVFVLAEYAAGIELPDEVFEHPIIQSMTEATNDLVTWSNDVFSYNKEQALGDTHNMITLLMAQHGLSLQGAVDFVGQLCAASITRFESGRTTLPSWGPDVDCDVQKYVMGLQDWIAGSLHWSFETERYFGKRGKEVRQAGVVKLSPMKAPKKV

>Copu6

MLQLPSHFVLQDLCAISGRACELKVSPLQREAGALATKWFDSIGVYDEIKFTKFTKFGKFDLFAALSFPEADLRHLETCLMFFFWAFSTDDLSDEGALQNRPDEVQAGHDVSNAVIDHPEAPRPAYPYAAMLYDLLERFRETGTEGAYARFIRAFEDWSESQVQQSQNRSEDRMPSIHEFILMRRATIGGAMVEAMIEYSLDIDLPDFIFEHPTIIAMSEATNDIMTWPNDLCSFNKEQADGDYQNLVFIIMEERGVGLQEGIDILTDMLSQRVDDYLALKASLPSFGPKVDYELARYLKALEHFTQGTVLWYYLSPRYFRTVDVSNRHNLVVPLFPQSFH

>Copu7

MPNRFYLPDTMVTWPWKTRTNPHADEVEEKCVEWCNAFPMMTKAYKKLKPDHLLVVSLFVLVIILVDDCTDAENADVARKTADLVKDAFEHSDQPRPAGEGPIGEIVRRFWQFSIGVITANVQVAFLKHFDEFLDSIVTQAGQRDEDVRLSVDKYLKLRRDNVGVMPFFPFLRTTSGPDLPEEIWDSAVIAEMTGHIIDMYIFDNDTISYGREYALGDTGHNIVTLLMQEHGIDVGSAVAWATARHAAAQKAFKDGLERLPSLGSHADAQVKEYLNGLGYWIRAYHVWSFKIERYFDGRGDEVKASRLVQLKPPASSVQSSAH

>Copu9

MSPTATFTTTSSEENAPTKFILPDLVSDCTYPLLLNDNCEPVARASEQWLIAGARLQEPRRTKFMGLLAGELTAACYPHADASHLRVCVDFMNWLFNMDDWLDDFDVDDTWGMRHCCLGAFRDPVGFETDKLGGLMSKSFFSRFRQDGGPGCTERFIHTMDLFFIAVAQQAGDRANGITPDLESYITVRRDTSGCKPCFALIEYAAGIDLPDHVIYHPTLAAMEEATNDLVTWSNDIFSYNKEQVTDDTHNMIPVLMRERGLDLQGAVDFVGRLCKGTIERFETERARLPSWGPELDAQVQTYIEGLQNWIVGSLHWSFDSHRYFGKDGHAVKKHRIVKLLPKRVPQQA

>Copu10

MSPSPTRFYLPDTMASWPWKTRTNPHADEVGGGDASSGHLSLLSESFSRLIQLVHSFDHLLVVSLFVLVIILVDDCTDTENADVARQTADLVNDAFEHSDQPRPAGEGPIGEIVRRFWQYAIGIISPKVQVAFLKHFGEFLESIVTQAGQRDEDVRLNVDTYLKLRRDNVGVMPFFPFLRPTSGPDLPEEIWESAVIAEMTGHIVDMYIFDNDTIFYGREYALGDTGHNIVTLLMQEYGIDVGSVAWATARHAAAQKAFKDGLERLPSHGAQADAQVKEYLNGLGYWIRAYHVWSFKIERYFDGRGDEVKVSRLVQLKPQ

>Copu11

MTSWPWKTKVNPYANAVRQRCAEWCSSFPMMAKAYKKLDPNQLLVVSLFSLVIVLVDDSTDVEDAAAAQQTATLVRDALQHPHQARPFGEAPIGEIVRRFWQLAIQTTHVDVQSAFLTHFDAFLESVVAQAGFRDHDSQLSIDAYLTIRRDTVGVMPFFPFLRPSADDRATENVWNSPIIAELTGYIVDMYIYDNDTISYAREHALGDIGHNIITLLMRDLNIDLGSAVSWAVMQHAVAQRAFIEGIARLPSWDADVDRQVRDYLDGLGHWAKAYHTWAFEVERYFGDRGKEVKATGLVRLRRS

## Fig. S2. Protein sequence analysis with Copu3 as reference

DDXXD motif presented in salmon, NSE-triad in yellow and WxxxxxRY motif in blue; effector triad R, D and S in green

CLUSTAL O(1.2.4) multiple sequence alignment

copu3 ----------MSATPAPTEFILPNLFSVC--PLTFGRSNPYYDEVIPEARAWIAKYNPFV

copu5 -----------MHLPEPFHFLLPDFSSHC--SYPLRLNK-HCVAAAAASEDWLIRLAQLR

copu6 ------------MLQLPSHFVLQDLCAISGRACELKVS-PLQREAGALATKWFDSIGVYD

copu7 ---------------MPNRFYLPDTMVTW--PWKTRTN-PHADEVEEKCVEWCNAFPMMT

copu9 MSPTATFTTTSSEENAPTKFILPDLVSDC--TYPLLLND-NCEPVARASEQWLIAGARLQ

copu10 ------------MSPSPTRFYLPDTMASW--PWKTRTN-PHADEVGGGDASS-GHLSLLS

copu11 -------------------------MTSW--PWKTKVN-PYANAVRQRCAEWCSSFPMMA

. .

copu3 DSKR----AEFVQ-GCNELLCSRVWPYAGREEFRTCCDFVNLLFVLDELSDDMGGADAR-

copu5 SPRNGRKLKKFMG-LKAGYLTALCYPDCPRTELRVVSDYMNFLFTLDDWSDEFAEAGVR-

copu6 EIKF----TKFTKFGKFDLFAALSFPEADLRHLETCLMFFFWAFSTDDLSDEGALQNRPD

copu7 KAYK-----K-----------------LKPDHLLVVSLFVLVIILVDDCTDAENADVAR-

copu9 EPRR----TKFMG-LLAGELTAACYPHADASHLRVCVDFMNWLFNMDDWLDDFDVDDTW-

copu10 ESFS-----RLIQ-L-----------VHSFDHLLVVSLFVLVIILVDDCTDTENADVAR-

copu11 KAYK-----K-----------------LDPNQLLVVSLFSLVIVLVDDSTDVEDAAAAQ-

. . .: . : . *: *

copu3 ---STCDSFIRVLNDPDAP--DT-SLIAQMTREFRARVAERAKPGCLRRFIALCGTYVEA

copu5 ---GLEQCVMGMLYDPT-V--KTDKAAGRLARSFWLRMIRTAGPRVQHRFIVAFEDFFRA

copu6 EVQAGHDVSNAVIDHPE--APRPAYPYAAMLYDLLERFRETGTEGAYARFIRAFEDWSES

copu7 ---KTADLVKDAFEHSDQPRPAGEGPIGEIVRRFWQFSIGVITANVQVAFLKHFDEFLDS

copu9 ---GMRHCCLGAFRDPVGF--ETDKLGGLMSKSFFSRFRQDGGPGCTERFIHTMDLFFIA

copu10 ---QTADLVNDAFEHSDQPRPAGEGPIGEIVRRFWQYAIGIISPKVQVAFLKHFGEFLES

copu11 ---QTATLVRDALQHPHQARPFGEAPIGEIVRRFWQLAIQTTHVDVQSAFLTHFDAFLES

: . :

copu3 VCVEAELREQGRVLDLRSFILLRRENSAVRCCLALAEYALGLELPDAVFNDPAFQSVYFC

copu5 VEQQSRDRAKGVMPDLESYIALRRDTSGCRPVFVLAEYAAGIELPDEVFEHPIIQSMTEA

copu6 QVQQSQNRSEDRMPSIHEFILMRRATIGGAMVEAMIEYSLDIDLPDFIFEHPTIIAMSEA

copu7 IVTQAGQRDEDVRLSVDKYLKLRRDNVGVMPFFPFLRTTSGPDLPEEIWDSAVIAEMTGH

copu9 VAQQAGDRANGITPDLESYITVRRDTSGCKPCFALIEYAAGIDLPDHVIYHPTLAAMEEA

copu10 IVTQAGQRDEDVRLNVDTYLKLRRDNVGVMPFFPFLRPTSGPDLPEEIWESAVIAEMTGH

copu11 VVAQAGFRDHDSQLSIDAYLTIRRDTVGVMPFFPFLRPSADDRATENVWNSPIIAELTGY

.: :: :** . . : . : . : : : :

copu3 AADMVCWSNDVYSYNMEQAKGHTGNNVVTVLMQEHGIDLQAAADRVGEVFGQLMEHYTSG

copu5 TNDLVTWSNDVFSYNKEQALGDTH-NMITLLMAQHGLSLQGAVDFVGQLCAASITRFESG

copu6 TNDIMTWPNDLCSFNKEQADGDYQ-NLVFIIMEERGVGLQEGIDILTDMLSQRVDDYLAL

copu7 IIDMYIFDNDTISYGREYALGDTGHNIVTLLMQEHGIDVGSAVAWATARHAAAQKAFKDG

copu9 TNDLVTWSNDIFSYNKEQVTDDTH-NMIPVLMRERGLDLQGAVDFVGRLCKGTIERFETE

copu10 IVDMYIFDNDTIFYGREYALGDTGHNIVTLLMQEYGIDVGS-VAWATARHAAAQKAFKDG

copu11 IVDMYIYDNDTISYAREHALGDIGHNIITLLMRDLNIDLGSAVSWAVMQHAVAQRAFIEG

*: : ** : * . .. *:: ::* : :.: :

copu3 SRSLPTWGGKVDADAARFLEAAGQWVVGNLEWSFETPRYFGPDHDEVRDTHRVLLK----

copu5 RTTLPSWGPDVDCDVQKYVMGLQDWIAGSLHWSFETERYFGKRGKEVRQAGVVKLSPMKA

copu6 KASLPSFGPKVDYELARYLKALEHFTQGTVLWYYLSPRYFRTVDVSNRHNLVVPLFPQSF

copu7 LERLPSLGSHADAQVKEYLNGLGYWIRAYHVWSFKIERYFDGRGDEVKASRLVQLKPPAS

copu9 RARLPSWGPELDAQVQTYIEGLQNWIVGSLHWSFDSHRYFGKDGHAVKKHRIVKLLPKRV

copu10 LERLPSHGAQADAQVKEYLNGLGYWIRAYHVWSFKIERYFDGRGDEVKVSRLVQLKPQ--

copu11 IARLPSWDADVDRQVRDYLDGLGHWAKAYHTWAFEVERYFGDRGKEVKATGLVRLRRS--

**: . . * : :: . : . * : : *

copu3 -------

copu5 PKKV---

copu6 H------

copu7 SVQSSAH

copu9 PQQA---

copu10 -------

copu11 -------

Fig. S3. Multiple sequence alignment of Copu9, Copu5, GME3638 and BvCS with Copu9 as reference

DDXXD motif presented in salmon, NSE-triad in yellow and WxxxxxRY motif in blue; effector triad R, D and S in green

cov pid 1 [ . . . . : . . . 80

1 Copu9 100.0% 100.0% --------------MSPT-ATFTTTSSEENAPTKFILPDLVSDCTYPLLLNDNCEPVARASEQWLIAGARLQEPRR----

2 Copu5 96.6% 52.7% --------------------------MHLPEPFHFLLPDFSSHCSYPLRLNKHCVAAAAASEDWLIRLAQLRSPRNGRKL

3 GME3638 95.4% 58.3% -----------------------------MRARSFILPDLVSDCPYTLRCNSNCEAVARASEAWMLEDANLSPKRR----

4 BvCS 99.7% 41.4% MSTASSPSLVASEIDSPHHSRTSSPSPTLSPPTSFILPDLVSHCNFPLTYHPAGDEQAAASLAWMLSFVPHFTPK---KV

cov pid 81 . 1 . . . . : . 160

1 Copu9 100.0% 100.0% TKFMGLLAGELTAACYPHADASHLRVCVDFMNWLFNMDDWLDDFDVDDTWGMRHCCLGAFRDPVGFET----------DK

2 Copu5 96.6% 52.7% KKFMGLKAGYLTALCYPDCPRTELRVVSDYMNFLFTLDDWSDEFAEAGVRGLEQCVMGMLYDPT-VKT----------DK

3 GME3638 95.4% 58.3% DAFLRLRGGELTAACYPDTDEACLRVAADFLNFLFSLDDWSDEFSMEDTCGLAQCVMCVLHDPDDFQT----------EK

4 BvCS 99.7% 41.4% AAMNGLQAGELTAYCYHDCPPERLRVVDDFMNYLFHLDNISDGMMAKNTTQLADWVMNAFEWPEKFQPTVNADGEVVEEI

cov pid 161 . . . 2 . . . . 240

1 Copu9 100.0% 100.0% LGGLMSKSFFSRFRQDGGPGCTERFIHTMDLFFIAVAQQAGDRANGITPDLESYITVRRDTSGCKPCFALIEYAAGIDLP

2 Copu5 96.6% 52.7% AAGRLARSFWLRMIRTAGPRVQHRFIVAFEDFFRAVEQQSRDRAKGVMPDLESYIALRRDTSGCRPVFVLAEYAAGIELP

3 GME3638 95.4% 58.3% AAGKLAKSFFNRFRQTAGPRCTRRFIDSMDLFFHAIAQQAQDRASGSAPSLEEYVALREDTSGCKPCFALIEYAAGMDLP

4 BvCS 99.7% 41.4% AAVKLARDYWSRCIQQAKPGVQQRFKSSMNMFFQAVEQQTNDRDGQVVPDLESYIDMRRDTSGCKPVFDLIEYALGFELP

cov pid 241 : . . . . 3 . . 320

1 Copu9 100.0% 100.0% DHVIYHPTLAAMEEATNDLVTWSNDIFSYNKEQVTDDTHNMIPVLMRERGLDLQGAVDFVGRLCKGTIERFETERARLPS

2 Copu5 96.6% 52.7% DEVFEHPIIQSMTEATNDLVTWSNDVFSYNKEQALGDTHNMITLLMAQHGLSLQGAVDFVGQLCAASITRFESGRTTLPS

3 GME3638 95.4% 58.3% DHVAHHPTITALEREANACISWSNDLFSYNVEQARGDTHNMIAVIMREDGRSLQEAVEYLGALCKLCIVHFEENRAMLPS

4 BvCS 99.7% 41.4% EEVVDHPVIKALNQDANDLVTWSNDVFSYNVEQARGDTHNMICIFMEHDGCTLQEAIDRVGGLCKQTIDAFVENKARVPS

cov pid 321 . . : . . . . ] 396

1 Copu9 100.0% 100.0% W---GPELDAQVQTYIEGLQNWIVGSLHWSFDSHRYFGKDGHAVKKHRIVKLLPKRVPQQA---------------

2 Copu5 96.6% 52.7% W---GPDVDCDVQKYVMGLQDWIAGSLHWSFETERYFGKRGKEVRQAGVVKLSPMKAPKKV---------------

3 GME3638 95.4% 58.3% W---GPEIDGEVDRYVLGLQDWMVGALHWSFDTARYFGDEGPAIKKHGVVTLLPRKSSS-----------------

4 BvCS 99.7% 41.4% FAHLGPEVDAWTTGYVQGLQDWIVGSLHWSFMTKRYFQEAGAEVKKTRFVKLLPIEEGRHKHIPPIYASAMVAATA

## Terpene structure elucidation: NMR Results

- Copu9: ^1^H

Fig. S4. ^1^H NMR spectrum of (+)-δ-cadinol produced by Copu9; Standardised on solvent (CDCl_3_) peak: ^1^H = 7.26 ppm, ^1^H NMR (500 MHz, CDCl_3_) δ 5.51 (dq, J = 5.3, 1.6 Hz, 1H), 1.98 (m, J = 13.9, 10.4, 6.2 Hz, 4H), 1.92 – 1.85 (m, 1H), 1.66 (s, 3H); 1.63 – 1.45 (m, 5H), 1.35 – 1.25 (m, 1H), 1.29 (s, 3H), 1.09 (qd, J = 13.2, 4.2 Hz, 1H), 0.88 (d, J = 6.9 Hz, 3H), 0.81 (d, J = 6.9 Hz, 3H).

- Copu9: ^13^C

Fig. S5. ^13^C NMR spectrum of (+)-δ-cadinol produced by Copu9; Standardised on solvent (CDCl_3_) peak: ^13^C = 77.2 ppm; ^13^C NMR (126 MHz, CDCl_3_) δ 134.36, 124.61, 72.55, 45.55, 44.08, 36.77, 35.31, 31.14, 27.97, 26.41, 23.66, 21.70, 21.52, 18.51, 15.31.


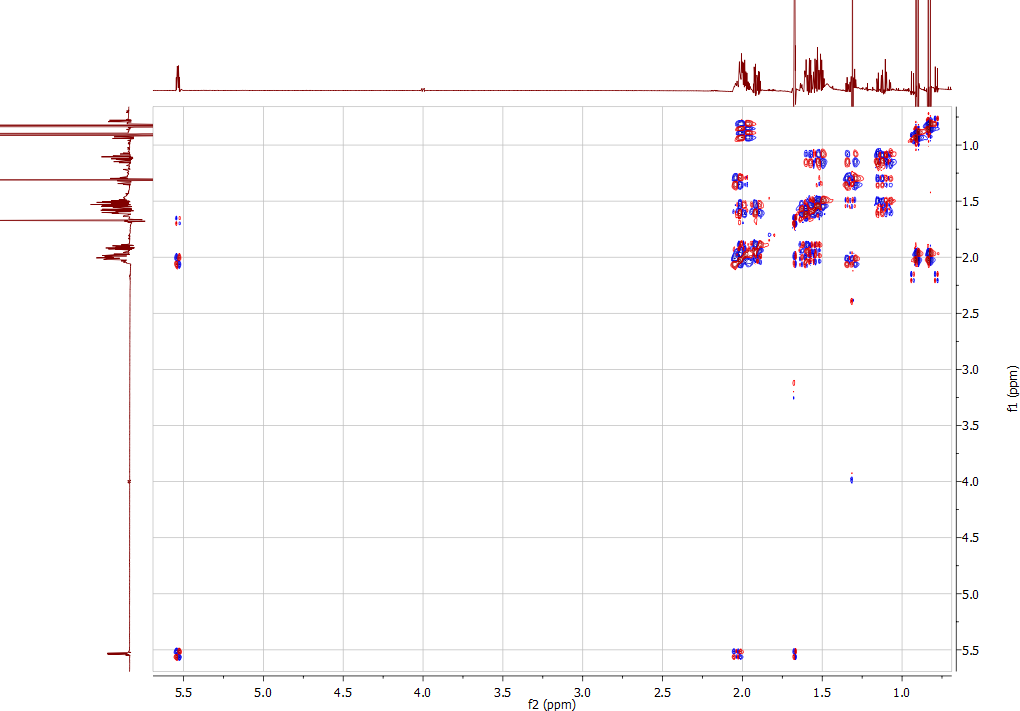


Fig. S6. ^1^H-^1^H COSY spectrum of (+)-δ-cadinol produced by Copu9


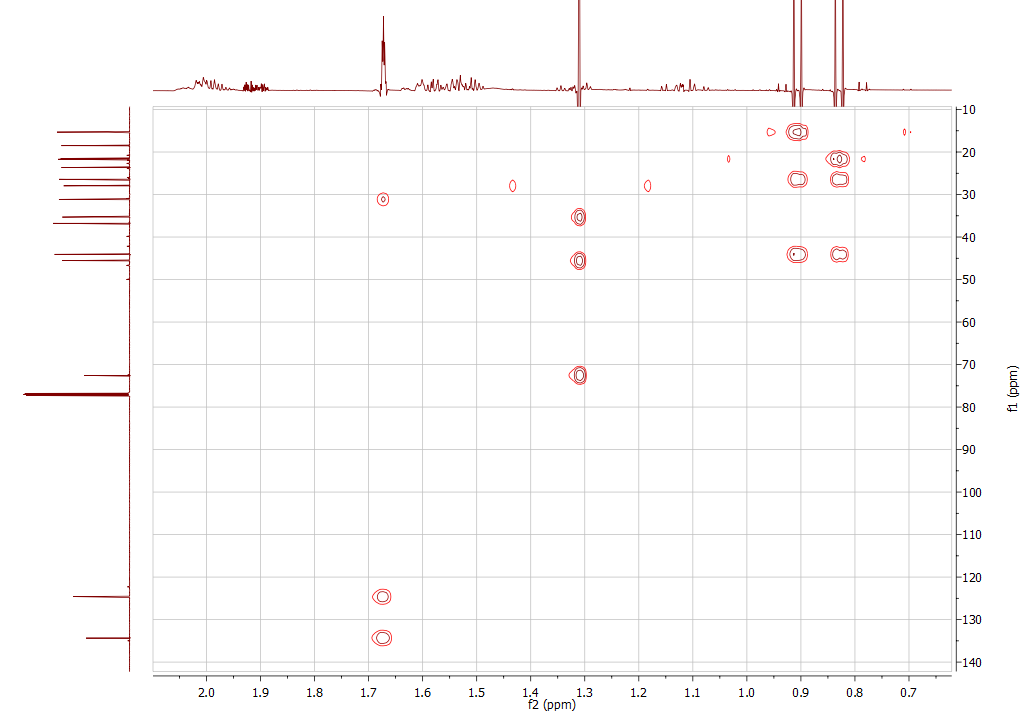


Fig. S7. ^1^H-^13^C HMBC spectrum of (+)-δ-cadinol produced by Copu9


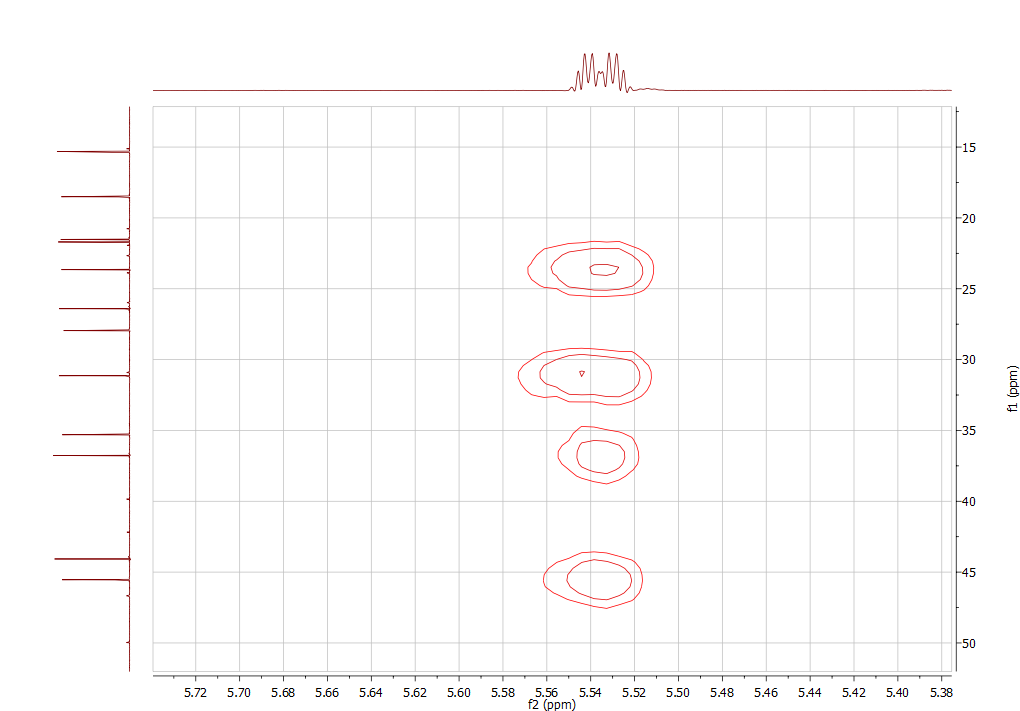


Fig. S8. ^1^H-^13^C HMBC spectrum of (+)-δ-cadinol produced by Copu9


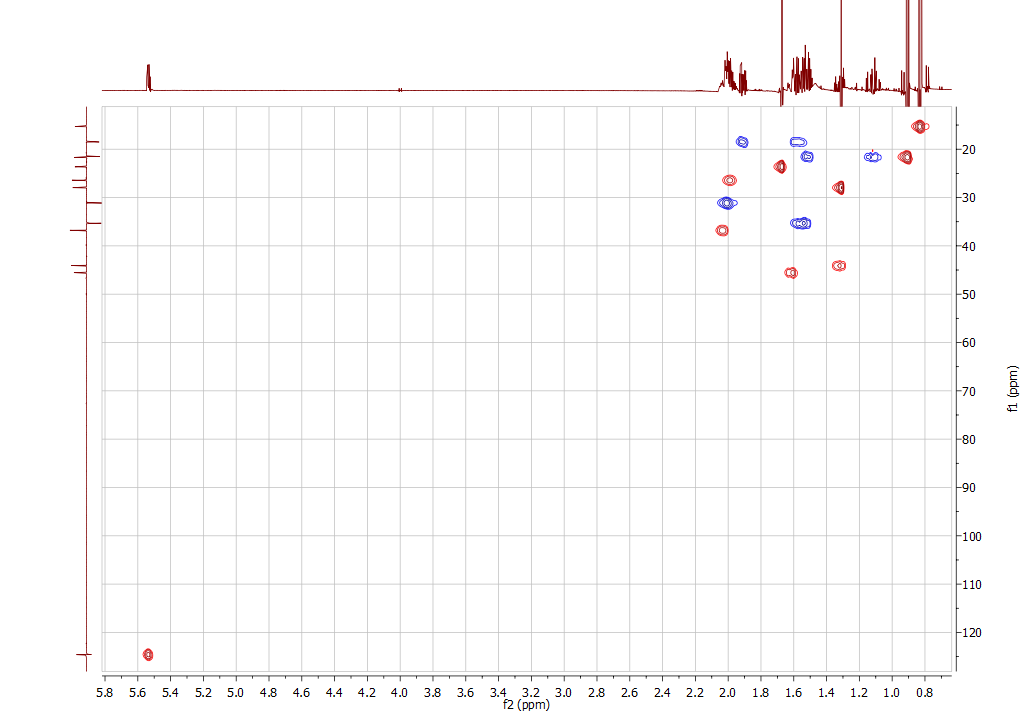


Fig. S9. ^1^H-^13^C HSQC spectrum of (+)-δ-cadinol produced by Copu9


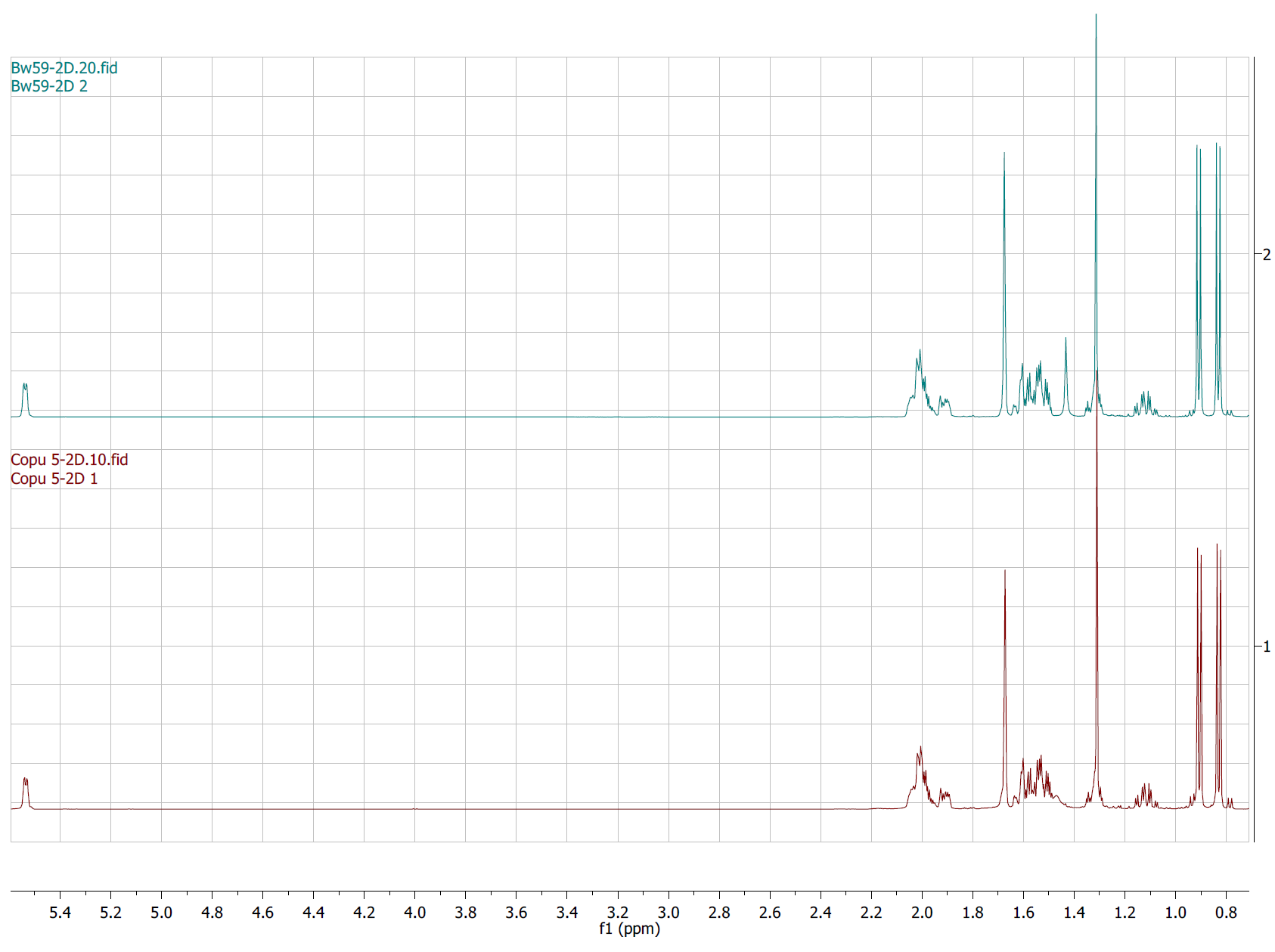


Copu5

Copu9

Fig. S10. Comparison of ^1^H-NMR spectra of (+)-δ-cadinol produced by Copu9 and Copu5


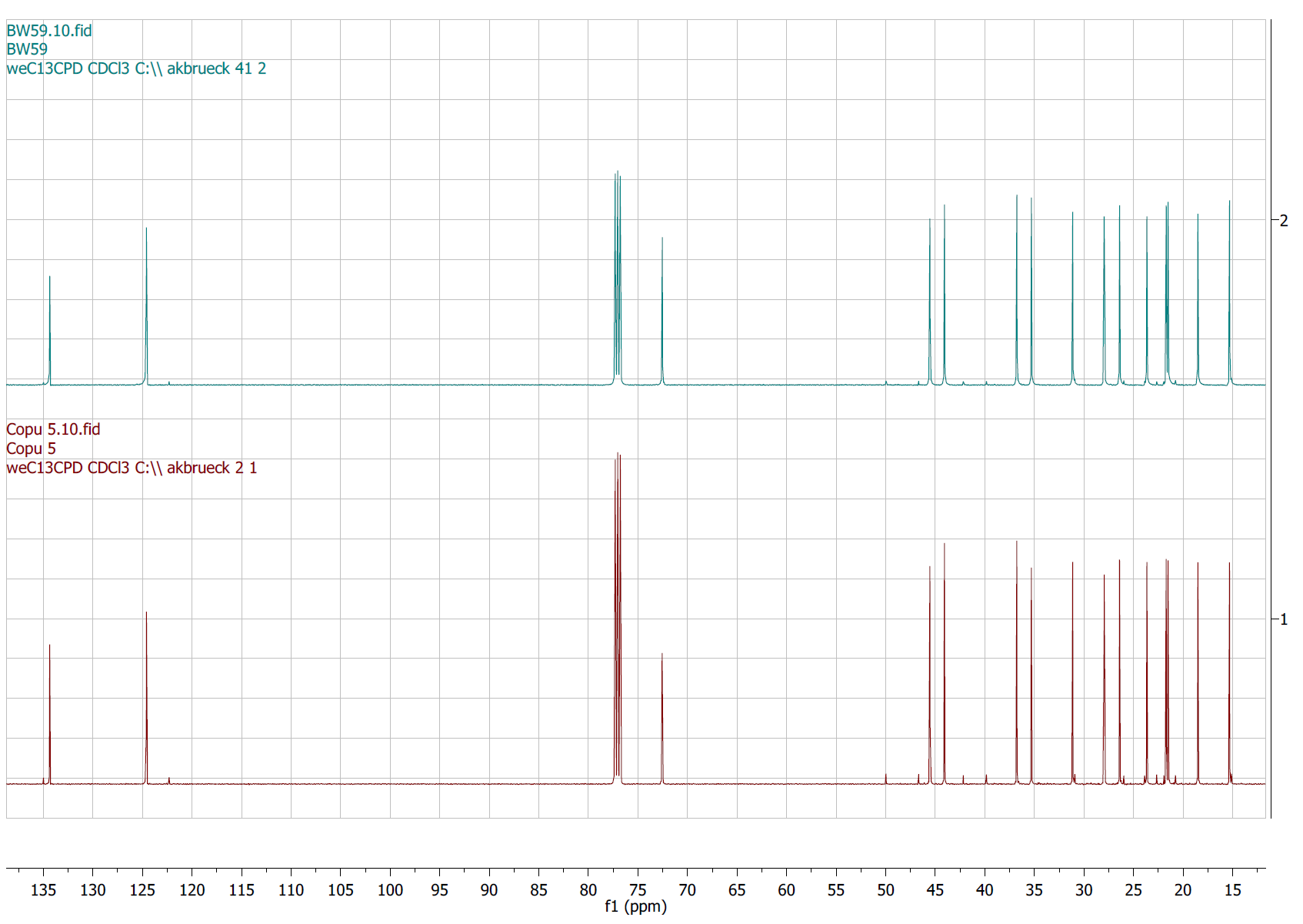


Copu5

Copu9

Fig. S11. Comparison of ^13^C-NMR spectra of (+)-δ-cadinol produced by Copu9 and Copu5


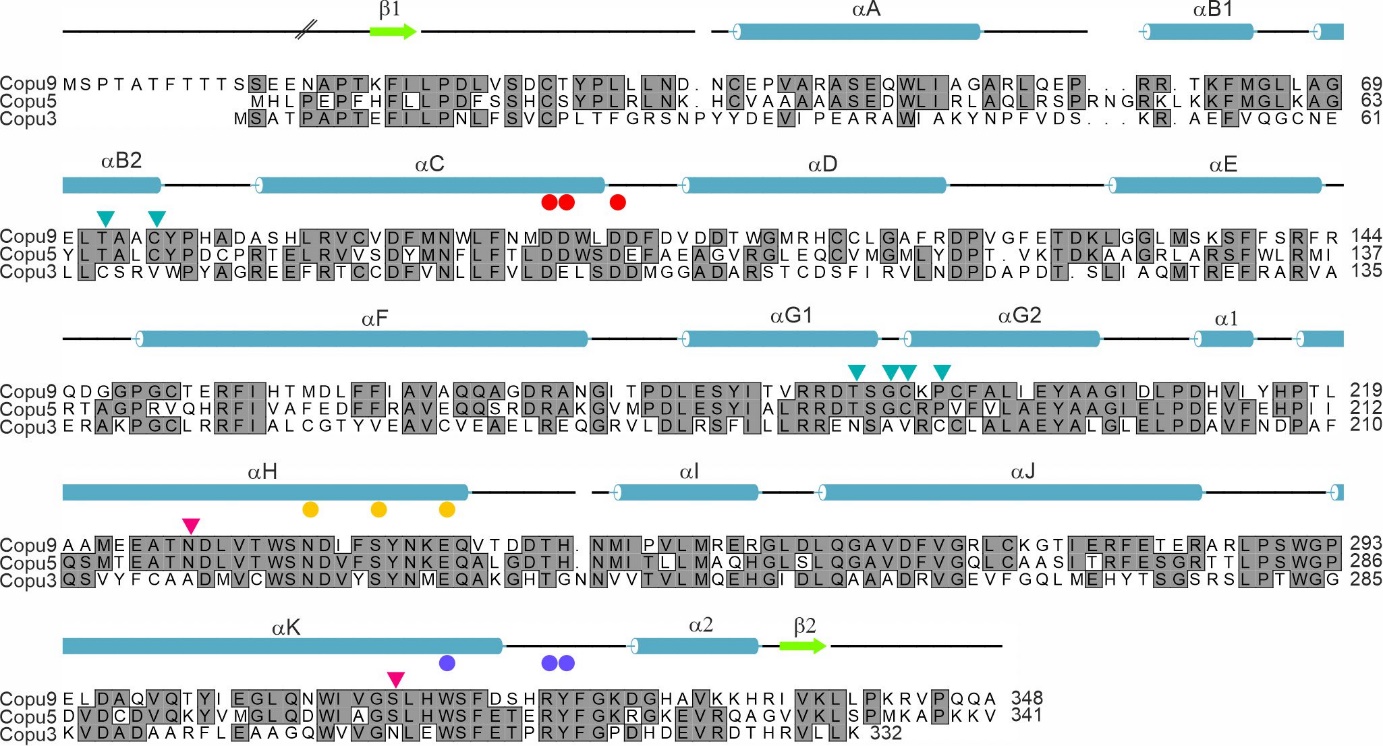


Fig. S12. Structure-based sequence alignment of Copu9, Copu5 and Copu3. On top of the primary sequence of Copu9 the secondary structure elements are drawn. Slashed lines at the N-terminus indicate the terminal residue, which is included in the crystal structure. The aspartate-rich motif (^99^DDWLD^103^) is highlighted with red spheres and the ^218^NSE^236^ motif with yellow spheres. The ^317^WxxxxxRY^324^ motif (with x as any amino acid) is indicated by blue spheres. Copu9 residues subjected to side directed mutagenesis are highlighted with triangles. Residues that have been subjected to mutagenesis, without altering the product, are shown as teal triangles, whereas single residue exchanges with an effect on the product profile are drawn as magenta triangles.


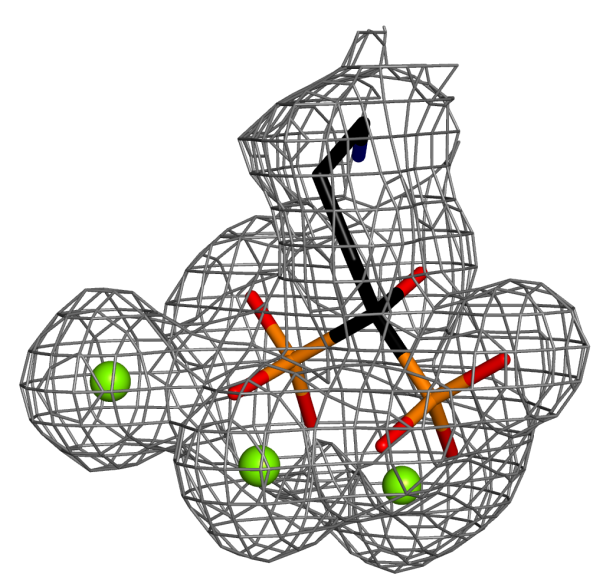


Fig. S13. Polder electron density map[2] shown as mesh at a σ-level of 2.0. AHD is presented as ball-stick-model with carbon atoms colored in black, oxygen in red, phosphorous in orange and nitrogen in light blue. Mg^2+^ ions are shown as green spheres.


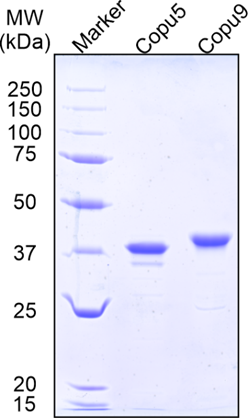


Fig. S 14: Sodium dodecylsulfate polyacrylamide gel electrophoresis (SDS-PAGE) of the purified Copu5 (theoretical mass including the His_6_-tag 41.9 kDa) and Copu9 (theoretical mass including the His_6_-tag 42.7 kDa) with Precision Plus unstained protein standard (Bio-Rad) on the left lane.


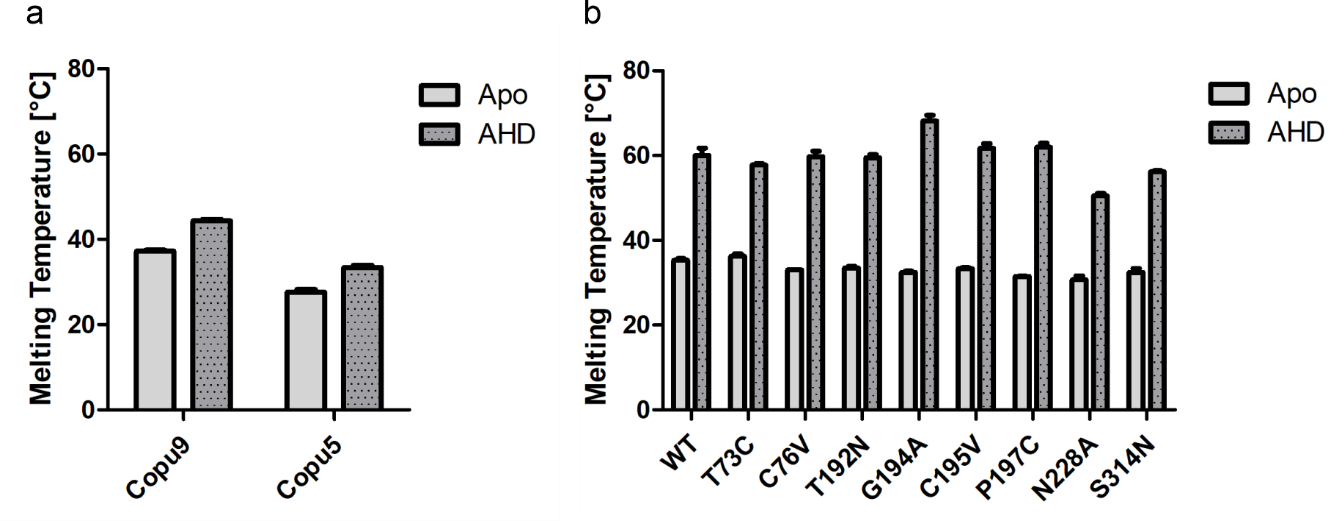


Fig. S15: Thermal shift analysis of Copu5 and Copu9 in pyrophosphate containing buffer with and without AHD (a) and Copu9 and its variants in Copu9 buffer with and without AHD

Table S1. Crystallographic data collection and model refinement statistics

| **Dataset** | Copu9•Mg^2+^_3_•AHD |
| --- | --- |
| PDB entry | 7OFL |
| **Data** **Collection** | |
| Wavelength [Å] | 0.9184 |
| Temperature [K] | 100 |
| Space group | *P*2_1_2_1_2 |
| Unit Cell Parameters  a, b, c [Å]  α, β, γ [°] | 74.45 76.62 135.11  90.0 90.0 90.0 |
| Resolution [Å]^a^ | 50.00 - 1.83  (1.94 - 1.83) |
| Reflections ^a^  Unique ^a^  Completeness [%]^a^  Multiplicity ^a^ | 68,244 (10,301)  98.6 (93.3)  7.3 (7.3) |
| Data quality ^a^  Intensity [I/σ(I)] ^a^  R_meas_ [%]^a ,b^  CC_1/2_ ^a,c^  Wilson B value [Å^2^] | 8.73 (1.01)  52.5 (302.0)  98.9 (44.0)  35.7 |
| **Refinement** | |
| Resolution [Å]^a^ | 50.00 - 1.83  (1.88 - 1.83) |
| Reflections ^a^  Number  Test Set [%] | 67671  3.1 |
| R_work_ [%]^a^  R_free_ [%]^a^ | 18.3 (34.9)  23.1 (36.5) |
| Asymmetric Unit  Protein: Residues, Atoms  Ligands: Molecules  Water molecules | 335 (A), 2,742 (A)  334 (B), 2,716 (B)  2 (AHD), 6 (Mg^2+^),  14 (EDO),  697 |
| Mean Temperature factors [Å^2^]^b^  All Atoms  Macromolecules  Ligands  Water molecules | 29.3  26.9 (A), 30.5 (B)  25.2 (AHD), 21.4 (Mg^2+^)  40.4 (EDO),  33.6 |
| RMSD from Target Geometry ^d^  Bond Lengths [Å]  Bond Angles [°] | 0.013  1.154 |
| **Validation Statistics** | |
| Ramachandran Plot ^f^  Residues in Allowed Regions [%]  Residues in Favored Regions [%]  Ramachandran plot *Z*-score ^f^ (RMSD)  whole  helix  sheet  loop  Molprobity Clashscore ^g^  Molprobity score ^f^ | 0.6  99.4  -1.20 (0.28)  -0.86 (0.19)  -  -0.04 (0.46)  3.48  1.16 |

^a^ data for the highest resolution shell in parenthesis

^b^ R_meas_(I) = ∑_h_ [N/(N-1)]^1/2^ ∑_i_ │I*_i_*_h_ - <I_h_>│ / ∑_h_∑_i_ I*_i_*_h_, in which <I_h_> is the mean intensity of symmetry-equivalent reflections h, I*_i_*_h_ is the intensity of a particular observation of h and N is the number of redundant observations of reflection h[3].

^c^ CC_1/2_ = (<I^2^> - <I>^2^) / (<I^2^> - <I>^2^) + σ^2^_ε_, in which σ^2^_ε_ is the mean error within a half-dataset[4].

^d^ RMSD – root mean square deviation

^e^ calculated with PHENIX [5]
^f^ calculated with MOLPROBITY [6]
^g^ Clashscore is the number of serious steric overlaps (> 0.4 ) per 1,000 atoms[6].

Table S 2: Results of a DALI [7] search with the coordinated of the structure of Copu9●Mg32+●AHD.

| **PDB ID** | **rmsd [Å]** | **sequence identity [%]** | **Z-score** | **protein** | **TPS family** | **Ligand in active site** | **resolution [Å]** | **Reference** |
| --- | --- | --- | --- | --- | --- | --- | --- | --- |
| 4okz | 1.7 | 23 | 39.1 | selinadiene synthase | sesquiterpene | dihydrofarnesyl, pyrophosphate | 1.90 | [8] |
| 4lz0 | 2.3 | 22 | 38.1 | Epi-isozizaene synthase | sesquiterpene | pyrophosphate, benzyl triethyl ammonium cation | 1.75 | [9] |
| 5dz2 | 1.9 | 24 | 36.8 | germacradienol/  geomisin synthase | sesquiterpene | alendronate | 2.11 | [10] |
| 5nx7 | 2.0 | 20 | 36.0 | pentalenene synthase | monoterpene | 2-fluoroneryl diphosphate | 1.51 | [11] |
| 6tjz | 2.1 | 25 | 36.0 | spiroviolene synthase | diterpene | none | 2.40 | [12] |
| 5ivg | 2.1 | 19 | 34.4 | aristolochene synthase | sesquiterpene | farnesyl thiolodiphosphate | 1.95 | [13] |
| 6oh6 | 2.3 | 16 | 34.1 | labdane-relateded diterpene synthase | diterpene | pyrophosphate | 2.07 | [14] |

Table S 3: Superposition of the experimentally obtained crystal structure of Copu9●Mg^2+^_3_●AHD, with the models for Copu9 and Copu5, respectively. The root mean square deviation (rmsd) for aligned pairs of Cα-atoms is given in [Å]. The number of aligned residues is provided in parenthesis.

| **PDB ID** | **Copu9●Mg^2+^_3_●AHD** | **Copu9 (model)** | **Copu5 (model)** |
| --- | --- | --- | --- |
| **Copu9●Mg^2+^_3_●AHD** | - | 1.2 (317) | 1.1 (319) |
| **Copu9 (model)** |  | - | 0.8 (321) |
| **Copu5 (model)** |  |  | - |


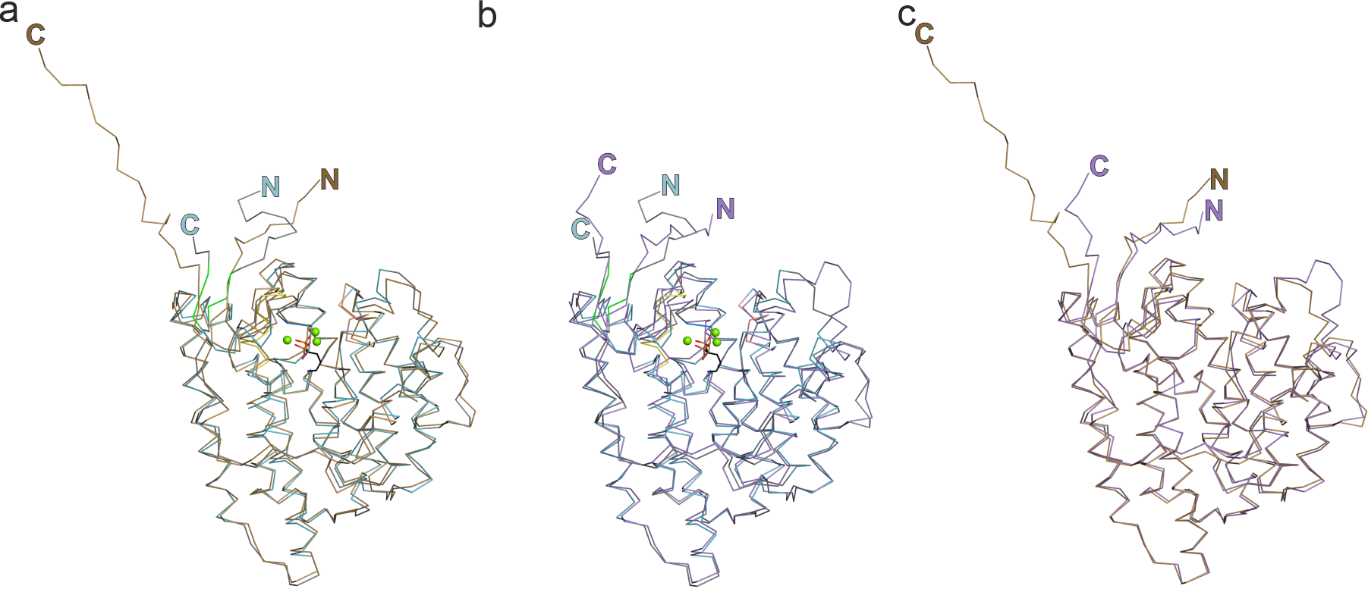


Fig. S 16: Comparison of the experimentally determined closed conformation of Copu9 with the modelled structures of Copu5 and Copu9. For clarity only one monomer is shown in ribbon representation. (a) Superposition **Copu9●Mg^2+^_3_●AHD** with α-helices are depicted in light blue and β-sheets in green. The Asp-rich motif is coloured in salmon and the NSE motif in yellow. The 3 Mg^2+^ ions are indicated as green spheres and alendronate in stick representation. The modelled structure of Copu9 is shown in brown, representing the open, inactive conformation. (b) Superposition of **Copu9●Mg^2+^_3_●AHD and the modelled Cop5 shown in violet.** (c) Superposition of the models of Copu5 (violet) and Copu9 (brown).

Table S4. Summary of active side residues in Copu3, Copu5 and Copu9. The listed residues are identical in Copu5 and Copu9. Only four of these residues are also identical in Copu3 (shown in grey). In the site directed mutagenesis study described the non-identical residues in Copu5 and Copu9 were replaced with the corresponding amino acids present in Copu3.

| **Copu3** | **Copu5** | **Copu9** | |  |
| --- | --- | --- | --- | --- |
| L63 | L65 | L72 | |  |
| C64 | T66C | | T73C | |
| V67 | C69V | | C76V | |
| L86 | L88 | L95 | |  |
| F87 | F89 | F96 | |  |
| N182 | T184N | | T192N | |
| A184 | G186A | | G194A | |
| V185 | C187V | | C195V | |
| C187 | P189C | | P197C | |
| A218 | N220A | | N228A | |
| W301 | W302 | W310 | |  |
| N305 | S306N | | S314N | |





Fig. S17. GC-Chromatograms of Copu5 WT and variants G186A, C187V, N220A and S306N; Detailed GC-MS analysis based on NIST database comparison allows for compound assignment of (1) tau-muurolene (RT: 15.51 min), (2) delta-cadinene (RT: 16.03 min), (3) cubebol (RT: 16.05 min), (4) (+)-δ-cadinol (RT: 17.66 min) and (5) α-cadinol (RT: 17.78 min); (*) represents the formation of a new side product germacrene-D-4-ol (RT: 16.85 min); G186A shows enhanced production of tau-muurolene; C187V shows enhanced production of δ-cadinene, cubebol and alpha-cadinol and formation of germacrene-D-4-ol; N220A shows a decrease in the formation of the main product, an increase in δ-cadinene and α-cadinol and the formation of germacrene-D-4-ol; S306N shows a decrease in the formation of the main product, an increase in δ-cadinene and α-cadinol and the formation of germancrene-D-4-ol.





Fig. S18. GC-Chromatograms of Copu9 WT and variants N228A and S314N; Detailed GC-MS analysis based on NIST database comparison allows for compound assignment of (1) tau-muurolene (RT: 15.51 min), (2) δ-cadinene (RT: 16.03 min), (3) cubebol (RT: 16.05 min), (4) (+)-δ-cadinol (RT: 17.66 min) and (5) α-cadinol (RT: 17.78 min); (*) represents the formation of a new side product germacrene-D-4-ol (RT: 16.85 min); N228A shows an increase in δ-cadinene and α-cadinol and the formation of germacrene-D-4-ol; S314N shows a decrease in the formation of the main product, an increase in δ-cadinene and α-cadinol and the formation of germancrene-D-4-ol.





Fig. S19. GC-MS spectrum of germacrene-D-4-ol (RT: 16.85 min) as identified by NIST database[15]; assigned with (*) in Fig. S 10 and Fig. S11.





Fig. S20. Proposed cyclication pathway of all products of Copu5 and Copu9 leading the carbocation reaction trajectory via the key carbocations germacryl and cadinyl[1, 16, 17].


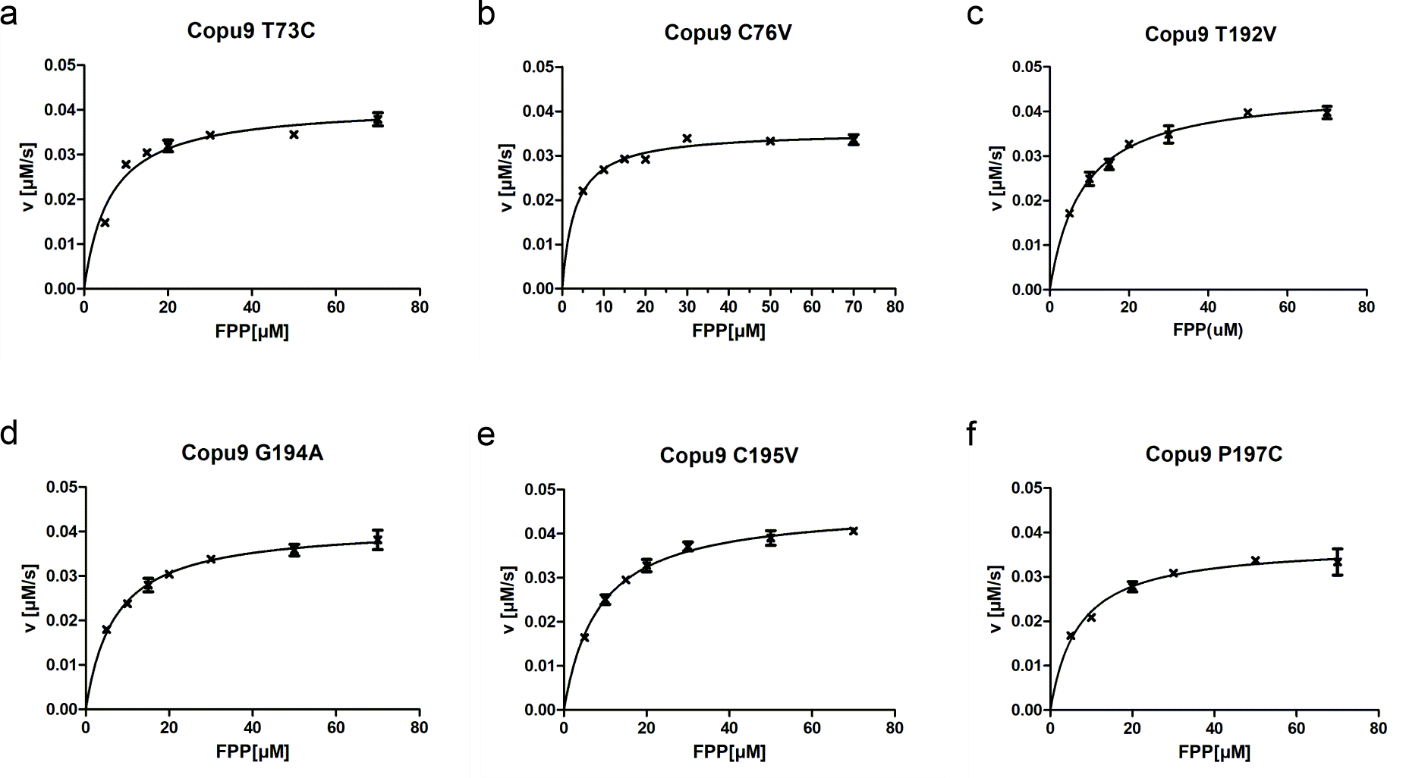


Fig S21: In vitro Michaelis-Menten kinetics of Copu9 variants T73C(a), C76V(b),T192N(c), G194A (d), C195V (e) and P197C (f) using the EnzChek^TM^ Pyrophosphate Assay. A non-linear regression analysis was performed on the data collected from the time-resolved steady-state kinetic assay.

References

1. Mischko W, Hirte M, Fuchs M, Mehlmer N, Brück TB. Identification of sesquiterpene synthases from the Basidiomycota Coniophora puteana for the efficient and highly selective β-copaene and cubebol production in E. coli. Microb Cell Fact. 2018;17:164. doi:10.1186/s12934-018-1010-z.

2. Liebschner D, Afonine PV, Moriarty NW, Poon BK, Sobolev OV, Terwilliger TC, Adams PD. Polder maps: improving OMIT maps by excluding bulk solvent. Acta Crystallogr D Struct Biol. 2017;73:148–57. doi:10.1107/s2059798316018210.

3. Diederichs K, Karplus PA. Improved R-factors for diffraction data analysis in macromolecular crystallography. Nat Struct Biol. 1997;4:269–75. doi:10.1038/nsb0497-269.

4. Karplus PA, Diederichs K. Linking crystallographic model and data quality. Science. 2012;336:1030–3. doi:10.1126/science.1218231.

5. Adams PD, Afonine PV, Bunkóczi G, Chen VB, Davis IW, Echols N, et al. PHENIX: a comprehensive Python-based system for macromolecular structure solution. Acta Crystallogr D Biol Crystallogr. 2010;66:213–21. doi:10.1107/S0907444909052925.

6. Williams CJ, Headd JJ, Moriarty NW, Prisant MG, Videau LL, Deis LN, et al. MolProbity: More and better reference data for improved all-atom structure validation. Protein Science. 2018;27:293–315. doi:10.1002/pro.3330.

7. Holm L. DALI and the persistence of protein shape. Protein Science. 2020;29:128–40. doi:10.1002/pro.3749.

8. Baer P, Rabe P, Fischer K, Citron CA, Klapschinski TA, Groll M, Dickschat JS. Induced-fit mechanism in class I terpene cyclases. Angew Chem Int Ed Engl. 2014;53:7652–6. doi:10.1002/anie.201403648.

9. Li R, Chou WKW, Himmelberger JA, Litwin KM, Harris GG, Cane DE, Christianson DW. Reprogramming the chemodiversity of terpenoid cyclization by remolding the active site contour of epi-isozizaene synthase. Biochemistry. 2014;53:1155–68. doi:10.1021/bi401643u.

10. Harris GG, Lombardi PM, Pemberton TA, Matsui T, Weiss TM, Cole KE, et al. Structural Studies of Geosmin Synthase, a Bifunctional Sesquiterpene Synthase with αα Domain Architecture That Catalyzes a Unique Cyclization-Fragmentation Reaction Sequence. Biochemistry. 2015;54:7142–55. doi:10.1021/acs.biochem.5b01143.

11. Karuppiah V, Ranaghan KE, Leferink NGH, Johannissen LO, Shanmugam M, Ní Cheallaigh A, et al. Structural Basis of Catalysis in the Bacterial Monoterpene Synthases Linalool Synthase and 1,8-Cineole Synthase. ACS Catal. 2017;7:6268–82. doi:10.1021/acscatal.7b01924.

12. Schriever K, Saenz-Mendez P, Rudraraju RS, Hendrikse NM, Hudson EP, Biundo A, et al. Engineering of Ancestors as a Tool to Elucidate Structure, Mechanism, and Specificity of Extant Terpene Cyclase. J Am Chem Soc. 2021;143:3794–807. doi:10.1021/jacs.0c10214.

13. Chen M, Chou WKW, Al-Lami N, Faraldos JA, Allemann RK, Cane DE, Christianson DW. Probing the Role of Active Site Water in the Sesquiterpene Cyclization Reaction Catalyzed by Aristolochene Synthase. Biochemistry. 2016;55:2864–74. doi:10.1021/acs.biochem.6b00343.

14. Centeno-Leija S, Tapia-Cabrera S, Guzmán-Trampe S, Esquivel B, Esturau-Escofet N, Tierrafría VH, et al. The structure of (E)-biformene synthase provides insights into the biosynthesis of bacterial bicyclic labdane-related diterpenoids. J Struct Biol. 2019;207:29–39. doi:10.1016/j.jsb.2019.04.010.

15. Linstrom P. NIST Chemistry WebBook, NIST Standard Reference Database 69: National Institute of Standards and Technology; 1997.

16. Yoshikuni Y, Martin VJJ, Ferrin TE, Keasling JD. Engineering cotton (+)-delta-cadinene synthase to an altered function: germacrene D-4-ol synthase. Chemistry & Biology. 2006;13:91–8. doi:10.1016/j.chembiol.2005.10.016.

17. Quin MB, Flynn CM, Schmidt-Dannert C. Traversing the fungal terpenome. Nat Prod Rep. 2014;31:1449–73. doi:10.1039/c4np00075g.
